# Ginger in supportive therapy against side effects of oncological treatment: a systematic review

**DOI:** 10.1186/s12906-026-05530-z

**Published:** 2026-08-18

**Authors:** Sarah Christina Burg, Jennifer Dörfler, Jens Büntzel, Judith Büntzel, Jutta Huebner

**Affiliations:** 1 Klinik für Innere Medizin II, Hämatologie und Internistische Onkologie, Universitätsklinikum Jena, Am Klinikum 1, Jena, 07747 Germany; 2 Klinik für Hals-Nasen-Ohren-Heilkunde, Südharz Klinikum Nordhausen, Dr.- Robert-Koch-Straße 39, Nordhausen, 99734 Germany; 3 Klinik für Hämatologie und Medizinische Onkologie, Universitätsmedizin Göttingen, Robert-Koch-Straße 40, Göttingen, 37075 Germany

**Keywords:** Ginger, Neoplasm, Complementary medicine, CINV, Emesis, Nausea

## Abstract

**Background:**

Oncology patients experience many side effects of their cancer treatment, which can affect their daily life. Phytotherapy is commonly used to reduce side effects or to support the treatment. Especially ginger plays a key role in the treatment of nausea and vomiting. The aim of this systematic review is to summarize the current scientific status of ginger in supportive therapy against side effects of oncological treatment.

**Methods:**

In January 2025 a systematic search was conducted searching five electronic databases (Embase, Cochrane, PsychInfo, CINAHL, Medline). Studies concerning the use, effectiveness and potential harm of ginger therapy on cancer patients were included. There was no time limit on the publication date of the studies. 25 publications concerning 24 studies with 2409 patients were included in this systematic review after removing publications that did not meet the inclusion criteria.

**Results:**

The patients were mainly diagnosed with breast cancer. The therapy concepts include ginger-capsules, -powder, -tea, and aromatherapy. Study outcomes were treatment associated symptoms, e.g. chemotherapy-induced nausea and vomiting, xerostomia, patients‘ nutrition, fatigue, and quality of life. The studies had low to high quality and reported heterogeneous results: some studies reported significant improved symptoms of chemotherapy-induced nausea and vomiting, xerostomia, nutrition, fatigue, and quality of life, while other studies did not find any changes concerning these endpoints. Furthermore, two studies found disadvantages of ginger concerning chemotherapy-induced nausea and vomiting.

**Conclusions:**

Ginger can be considered a promising candidate for complementing the supportive therapy against the side effects of cancer treatment of oncological patients. The data on this is still limited due to heterogeneous results and methodical limitations of the included studies.

**Trial registration:**

As this is a systematic review, clinical trial number is not applicable.

**Supplementary Information:**

The online version contains supplementary material available at 10.1186/s12906-026-05530-z.

## Background

An estimated 19.96 million patients worldwide were diagnosed with cancer in 2022 [1]. For these patients, the subsequent cancer therapy is a very distressing time [2]. In addition to the final outcome, the negative symptoms associated with cancer therapy play a major role for the patients. Sufficient supportive care alleviating side effects is necessary to ensure successful completion of oncological treatment [3]. In patients undergoing chemotherapy, chemotherapy-induced nausea and vomiting (CINV) plays a major role in terms of adverse effects [4]. Thanks to modern antiemetics such as 5-hydroxytryptamine 3 receptor- (5-HT3) and neurokinin-1 receptor- (NK-1) antagonists, the control of CINV has been significantly improved [5]. However, despite these modern therapies, about 30–40% of patients still suffer from CINV [6]. Some patients turn to phytotherapeutics to support conventional oncological therapy or to alleviate its side effects [7]. Various reviews investigated the use of phytotherapeutics in cancer therapy [8]. In the review by Zadorozhna et al. [9] the influence of ginger on tumor cells via various pathways, including cell cycle arrest and the inhibition of angiogenesis, was demonstrated. Similar results were obtained in relation to breast cancer in the review by Usman et al. [10] and in relation to colon cancer in the review by Xiang et al. [11]. The mentioned reviews remain at a preclinical level, meaning that the effect has only been proven in relation to cell series or animal models, not in clinical studies. In clinical studies various phytotherapeutic agents have been identified as a supplementary option in supportive therapy against the side effects of cancer therapy. The review by Ruan et al. [12] demonstrated a positive influence of black cohosh on neurovegetative and psychological complaints during breast cancer therapy, while the study by Karbasforooshan et al. [13] showed a positive effect of topical silymarin in the prevention of acute radiodermatitis in breast cancer therapy. Especially regarding nausea and vomiting, ginger plays a central role. Ginger has been used for medicinal purposes since ancient times [14]. Today, ginger is already used successfully for nausea in other areas such as pregnancy [15] or motion sickness [16]. The review by Marx et al. [17] analyzed the possible mechanisms by which ginger could influence CINV based on preclinical studies, finding various pathways through which ginger could have a positive influence on CINV. Ginger has several biologically active components, especially gingerols and shogaols. They have an influence on various mechanisms that are related to CINV, including an antagonistic effect on 5-HT3, substance P, and muscarinic receptors [18]. Ginger has also been shown to increase gastrointestinal motility and accelerate gastric emptying [19].

These multiple effects of ginger may have a positive influence on cancer patients’ symptom burden. Alleviating CINV but as well xerostomia (dry mouth), may translate into secondarily effects on nutrition, fatigue, and quality of life (QoL) in general and need to be evaluated clinically. There are already reviews investigating different aspects of the use of ginger to treat side effects of oncological therapy, mostly focusing on CINV among diverse patient populations. The review by Hardi et al. [20] demonstrated a positive effect of ginger on CINV in pediatric patients undergoing oncological therapy. The meta-analyses by Lin et al. [21] and by Kim et al. [22] for breast cancer patients also showed a positive effect on CINV. This systematic review aims to summarize the current scientific status of ginger in the supportive therapy against side effects of cancer treatment in general, without restrictions in relation to the type of cancer or endpoints of the studies, to provide an overall overview. The focus is on patient-relevant factors and the therapeutic safety of ginger intake in patients undergoing conventional cancer therapy.

## Method

### Criteria for including and excluding studies in the review

Inclusion and exclusion criteria are listed in Table 1 based on a PICO-model, which categorizes the criteria into five categories: patient, intervention, comparison, outcome, and others.

**Table 1:** Inclusion and exclusion criteria

PICO	Inclusion criteria	Exclusion criteria
Patient	Cancer patients (all entities and stages), Adult patients (age > 18)	Patients with only precancerous conditions or Carcinoma in situ, Preclinical studies, Study population with more than 20% children or precancerous conditions
Intervention	Every intervention with ginger	
Comparison	All possible control groups (placebo, standard care, observation)	Other study types (one-armed/non-controlled studies, case report or series)
Outcome	Primary endpoints were all patient-relevant symptoms/toxicities, secondary endpoints were response data, survival data, and quality of life	No patient- centred data, for example laboratory parameters
Others	Randomized controlled trials, Language: German and English, Full publication	Grey literature (conference articles, abstracts, letters, ongoing studies, unpublished literature…)

*PICO* Patient – Intervention – Comparison – Outcome

### Study selection

A systematic research was conducted using five databases (Medline (Ovid), CINAHL (EBSCO), EMBASE (Ovid), Cochrane CENTRAL and PsycINFO (EBSCO)) January 2025. For each of these databases a complex search strategy was developed consisting of a combination of MeshTerms, keywords, and text words in different spellings connected to cancer and ginger therapy (additional file 1, Table e1). The search string was restricted by filters of study or publication type, there was no time limit on the publication date. After importing the search results into EndNote 20.4, all duplicates were removed and a title-abstract-screening was carried out by two independent reviewers (SB and JD). When title and abstract did not have sufficient information for screening purposes, a full-text copy was retrieved. In case of disagreement, consensus was made by discussion or a third author was consulted (JH). After that, all full texts were retrieved and screened again independently by both reviewers. Additionally, bibliography lists of all retrieved articles and systematic reviews were searched for relevant studies. PRISMA guidelines [23] were followed.

### Assessment of risk of bias and methodological quality

All characteristics were assessed by two independent reviewers (SB and JD). In case of disagreement, a third reviewer was consulted (JH) and consensus was made by discussion.

The risk of bias (RoB) in the included studies was analyzed with the Cochrane revised Risk of Bias Tool 2.0 [24]. Additional criteria concerning methodology were size of population, application of power analysis, adequacy of statistical tests (e.g. control of premises or multiple testing), and selective outcome reporting (report of all assessed outcomes with specification of statistical data as the p-value).

### Data extraction

Data extraction was performed by one reviewer (SB) and controlled by two independent reviewers (JD and JH). As a template for data extraction, the evidence tables from the national Guideline on Complementary and Alternative Medicine in Oncological Patients of the German Guideline Program in Oncology (https://www.leitlinienprogramm-onkologie.de/english-language/) were used.

## Results

**Fig. 1:**
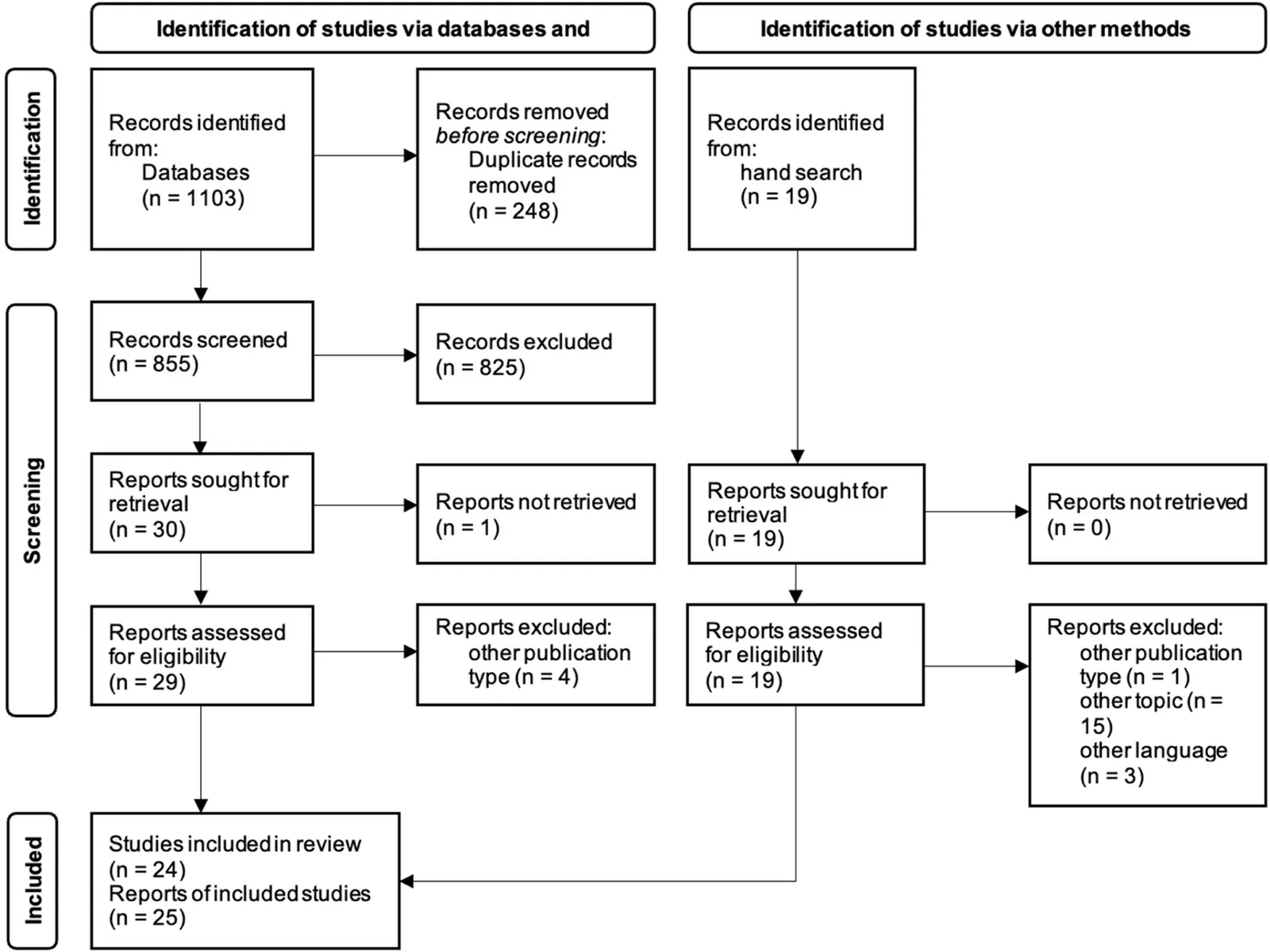
PRISMA Diagram

The systematic research revealed 1103 results. 19 studies were added by hand search. At first, duplicates were removed leaving 855 studies. After screening title and abstract, 30 studies remained to complete review (Fig. 1: PRISMA Diagram). Finally, 25 publications were analyzed in this review. Two publications reported different outcomes from one study [25, 26]. According to this, the 25 publications reported data from 24 relevant studies. Detailed characterization of the included studies may be seen in Table 2 and for additional information in additional file 1, Table e3.

**Table 2:** Characteristics of included studies

Reference	Sample size	Cth / Duration	Intervention (A)/ Control (B-D)	Endpoints	Outcomes
Ansari et al. (2016) [27]	*N* = 150 A = 75 B = 75	Cth: doxorubicin based Cth Duration: 3 cycles	A: ginger capsules 1 g/d B: placebo	1. Nausea (severity)	1: No sign. differences Subgroup analyses of different Cth-combinations: no sign. differences
				2. Vomiting (severity)	2: No sign. differences Subgroup analyses of different Cth-combinations: doxorubicin + cyclophosphamide: A < B sign. (*p* < 0.05)
				3. Adherence	3: A vs. B: 76% vs. 82%
Arslan et al. (2015) [28]	*N* = 60 A = 30 B = 30	Cth: anthracycline based Cth Duration: 2 cycles	A: powdered ginger 1 g/d B: no additional intervention	1. Nausea (severity)	1: Day 2–5, five day mean: A < B sign. (*p* < 0.001; *p* = 0.001; *p* = 0.001; *p* = 0.001; *p* < 0.001) Acute and delayed nausea severity: A < B sign. (*p* < 0.001)
				2. Vomiting (number of episodes)	2: Day 2, 3, 5, five day mean: A < B sign. (*p* = 0.01; *p* = 0.01; *p* = 0.04; *p* = 0.011)
				3. Retching (number of episodes)	3: No sign. differences
Bossi et al. (2017) [29]	*N* = 251 A = 125 B = 126	Cth: high-dose cisplatin Duration: 2 cycles	A: ginger capsules 0.16 g/d B: placebo	1. Nausea (incidence of (significant) delayed nausea)	1: Incidence of delayed nausea: in total no sign. differences (cycle 1: *p* = 0.851, cycle 2: *p* = 0.379). Subgroup analysis: lung cancer patients: A > B sign. (cycle 2: *p* = 0.042) Incidence of significant delayed nausea: in total no sign. differences (cycle 1: *p* = 0.163, cycle 2: *p* = 0.239). Subgroup analysis: men: A > B sign. (*p* < 0.05)
				2. Nausea (incidence of (significant) intercycle nausea)	2: Incidence of intercycle nausea: in total no sign. differences (cycle 1: *p* = 0.367, cycle 2: *p* = 0.417). Subgroup analysis: lung cancer patients: A > B sign. (in total; cycle 1) Incidence of significant intercycle nausea: in total no sign. differences (cycle 1: *p* = 0.074, cycle 2: *p* = 0.361). Subgroup analysis: men: A > B sign. (*p* < 0.05)
				3. Nausea (incidence of (significant) anticipatory nausea)	3: Incidence of anticipatory nausea: in total no sign. differences (cycle 1: *p* = 0.823, cycle 2: *p* = 0.629). Incidence of significant anticipatory nausea: in total no sign. differences (cycle 1: *p* = 0.666, cycle 2: *p* = 0.536).
				4. Nausea (intensity)	4: No sign. differences (*p* = 0.414–0.959)
				5. QoL impact of nausea on daily life (ability to maintain daily life))	5: In total no sign. differences. Subgroup analysis: women, head-neck-cancer patients: A > B sign. (*p* = 0.048; *p* = 0.038)
				6. Fatigue	6: No sign. differences
				7. Adverse events	7: In total no sign. differences. Treatment related side effects: A > B sign. Gastrointestinal events: A > B
Chenbing et al. (2023) [30]	*N* = 172 A = 43 B = 43 C = 43 D = 43	Cth: oxaliplatin regimes (150 / 200 mg) Drop out: 12 (3 in each group)	A: powdered ginger 2 × 500 mg per day (30 min before Cth) B: acupressure of P6 (anterior on the forearm, 3-finger widths up from the wrist between the tendons of the palmaris longus and flexor carpi radialis) (for 10 min (30 min before Cth) C: joint (acupressure and ginger as mentioned) D: no additional intervention	1. Nausea (acute (day 1), delayed (day 2–5))	**1**: Acute: A > B sign. (*p* = 0.037) No sig. differences (*p* = 0.574, *p* = 0.109). Delayed: A > C sign. (*p* = 0.001); A < D sign. (*p* = 0.000). No sign. differences between A and B (*p* = 0.142).
				2. Vomiting / retching (acute (day 1), delayed (day 2–5))	2: Acute vomiting: no sign. differences (*p* = 0.320, *p* = 0.112, *p* = 0.740). Delayed: no sign. differences (*p* = 0.153, *p* = 0.108, *p* = 0.187). Acute retching: A > B, C sign. (*p* = 0.013, *p* = 0.000). No sign. differences between A and D (*p* = 0.725). Delayed retching: A > B, C sign. (*p* = 0.005, *p* = 0.000); A < D sign. (*p* = 0.001).
				3. QoL: functional living index [31] (for nausea and vomiting: degree, activity, cooking, eating, drinking, social contact, daily living, personally difficulties)	3: Nausea: A > D sign. concerning the degree of nausea, activity, cooking, eating, drinking, and personal difficulties (*p* < 0,05). A < B sign. concerning the degree of nausea, activity, cooking, eating, drinking, social contact, daily living and personal difficulties (*p* < 0.05). A < C sign. concerning all categories (*p* < 0.05). Vomiting: No sig. difference between A and D (*p* > 0.05). A < B sign. concerning degree, activity, eating, drinking, social contact, daily living and personal difficulties (*p* < 0.05). A < C sign. concerning all categories (*p* < 0.05).
				4. Benefit finding	4: No sign. differences (*p* < 0.05).
				5. Treatment satisfaction	5: No sig. differences between A and the other groups (*p* < 0.05).
				6. Adverse events	6: No sign. differences (*p* < 0.05).
Crichton et al. (2024) [32]	*N* = 103 A = 52 B = 51	Cth: single day moderately to highly emetogenic Cth Duration: 3 cycles	A: ginger 4 × 300 mg per day (for 5 days per cycle) B: placebo	1. QoL (chemotherapy-induced nausea related: decline)	1: Sign. main effect for group (*p* = 0.003) and for time (*p* < 0.001). Cycle 2: A < B sign. (*p* = 0.002). Clinically meaningful for all cycles.
				2. QoL (vomiting- and CINV-related: decline)	2: Vomiting related QoL: main effect for group (*p* = 0.001) and interaction effect between group and time (*p* < 0.001). Cycle 2, 3: A < B sign. (*p* = 0.003, *p* < 0.001). CINV related QoL: main effect for group (*p* < 0.001). Cycle 2, 3: A < B sign. (*p* < 0.001, *p* = 0.001). Clinically meaningful for all cycles.
				3. QoL (health-related)	3: No sign. differences (*p* > 0.003).
				4. CINV (incidence and severity: anticipatory (one day before Cth), acute (12–24 h after Cth), delayed (4 days after Cth))	4: Incidence and severity of anticipatory and acute nausea: no sign. differences (*p* > 0.003). Incidence of delayed nausea: cycle 3: A < B sign. (*p* = 0.002). Clinically meaningful for cycle 2, 3. Severity of delayed nausea: main effect for group (*p* = 0.003). Cycle 2: A < B sign. (*p* = 0.002). Clinically meaningful for all cycles. No anticipatory vomiting reported at any cycle. Acute vomiting: no sign. differences (*p* > 0.003). Delayed vomiting: main effect for group (*p* = 0.003), time (*p* < 0.001) and interaction effect between group and time (*p* < 0.001) concerning number of episodes. Incidence at cycle 2, 3: A < B sign. (*p* = 0.001, *p* = 0.001), clinically meaningful.
				5. Fatigue	5: Main effect for group (*p* = 0.002), time (*p* = 0.001) and interaction effect between group and time (*p* < 0.001). Cycle 1: A < B sign. (*p* < 0.001), clinically meaningful.
				6. Nutrition status	6: Main effect for time for PG-SGA score (*p* < 0.001). Malnutrition: no sign. differences (*p* > 0.003), but lower incidence of malnutrition in A than B at cycle 3 was considered clinically meaningful.
				7. Mental health (depression and anxiety)	7: No sign. differences (*p* > 0.003).
				8. Adverse events	8: No serious adverse events related to the intervention. In total 19 mild and 1 moderate adverse event possibly related to the intervention. Reflux was more prevalent in A than in B (A: 18%, B:2%).
Fahimi et al. (2011) [33]	*N* = 50	Cth: cisplatin-based regimens Duration: 2 cycles	A: ginger capsules 1 g/d B: placebo	1. Nausea (prevalence, severity, duration: acute (day 1), delayed (day 2–3))	1: No sign. differences: prevalence (acute: *p* = 0.388; delayed: *p* < 0.999), severity (acute: *p* = 0.14; delayed: *p* = 0.73), duration (acute: *p* = 0.93; delayed: day 2: *p* = 0.59; day 3: *p* = 0.82)
				2. Vomiting (prevalence, severity, duration: acute (day 1), delayed (day 2–3))	2: No sign. differences: prevalence: (acute: *p* = 0.070; delayed: day 2: *p* = 0.687; day 3: *p* < 0.999), severity (acute: *p* = 0.14; delayed: day 2: *p* = 0.72; day 3: *p* = 0.78)
Kadhim et al. (2021) [34]	*N* = 60 A = 30 B = 30	Cth: not reported Duration: 1 cycle	A: ginger tea 1.5 g/d B: no additional intervention	1. Vomiting (acute, delayed)	1: No sign. differences (acute: *p* = 0.798; delayed: *p* = 0.779)
				2. Nausea (acute, delayed)	2: No sign. differences (acute: *p* = 0.182; delayed: *p* = 0.112)
				3. Nausea (severity: acute, delayed)	3: A < B sign. (acute: *p* = 0.007; delayed: *p* = 0.013)
Konmun et al. (2017) [35]	*N* = 94 A = 48 B = 46	Cth: moderately to highly emetogenic adjuvant Cth Duration: 3 cycles	A: 6-gingerol capsules 0.2 g/d B: placebo	1. Nausea (complete response rate (no emesis / rescue treatment) for all cycles (overall, acute (≤ 24 h), delayed (24–120 h)))	1: Complete response rate: overall, acute-, delayed phase: A > B sign. (overall: *p* < 0.001; acute: *p* = 0.003; delayed: *p* < 0.001)
				2. Nausea (complete response rate for cycle 1)	2: Complete response rate for cycle 1: overall, delayed: A > B sign. (overall: *p* = 0.001; delayed: *p* = 0.004) acute: no sign. differences (*p* = 0.057)
				3. Nausea, vomiting (severity)	3: All grade, grade 3 vomiting: A < B sign. (all grade: *p* < 0.001; grade 3: *p* < 0.001). No grade 4 vomiting was observed. Nausea: A < B sign. (mild, moderate, severe: *p* < 0.001)
				4. Tolerability	4: None of the patients was discontinued from the study due to toxicity. No dose reduction was required. 3 patients discontinued the study early: A: 1 patient because of grade 3 vomiting. B: 2 patients due to termination of Cth after cycle 2 / due to grade 2 dyspepsia.
				5. Nutrition (appetite score)	5: A < B sign. for day 22, 43, 64, adjusted with baseline values of day 1 (*p* = 0.001)
				6. QoL (health-related)	6: A > B sign. in mean change of the total score (*p* = 0.005), physical (*p* < 0.001) and emotional (*p* = 0.006) wellbeing. In mean change A is also clinically different to B. No sign. differences in social or family wellbeing (*p* = 0.203) and functional wellbeing (*p* = 0.147)
				7. Adverse events	7: In general no sign. differences (*p* = 0.244–1.000). Fatigue grade 3: A < B sign. (*p* = 0.020) No sign. adverse event related to 6-gingerol
				8. Adherence	8: Adherence: 98.4% (A: 99.1%; B: 97.7%)
Li et al. (2018) [36]	*N* = 146 A = 73 B = 73	Cth: cisplatin-based regimens Duration: 1 cycle	A: ginger capsules 0.5 g/d B: placebo	1. CINV (incidence, severity: acute)	1: No sign. differences in the incidence of acute nausea (*p* = 0.174) and vomiting (*p* = 0.309). No sign. differences in mean nausea scores (*p* = 0.246) and frequency of vomiting (*p* = 0.256) in acute phase; no differences even after controlling for intake of aprepitant
				2. CINV (incidence, severity: delayed)	2: No sign. differences in the incidence of delayed nausea (*p* = 0.214) and vomiting (*p* = 0.813). No sign. differences in mean nausea scores (*p* = 0.347) and frequency of vomiting (*p* = 0.718) in delayed phase
				3. QoL	3: No sign. differences (*p* = 0.884)
				4. Adherence	4: B: 5 participants took fewer capsules than the protocol required (6–8 out of 10)
				5. Success of blinding	5: Participants were likely to correctly guess their treatment arm (*p* = 0.01); given reasons: taste of ginger (A: 42.3%; B: 27.5%; *p* = 0.068), effect of the capsule (A: 21.1%, B: 10.1%; *p* = 0.103)
				6. Adverse events	6: No sign. differences: drowsiness (*p* = 0.163), dry mouth (*p* = 0.086), heartburn (*p* = 0.494), flushing (*p* = 0.184); no study- treatment-related adverse event
Lua et al. (2015) [25] Salihah et al. (2016) [26]	*N* = 75 A = 38 B = 37	different Cth agents (on highly emetogenic Cth = 86.7%; low emetogenic = 13.3%) Duration: 2 cycles	A: aromatherapy necklace filled with 2 drops ginger essential oil B: fragrance-matched artificial placebo	Lua:	
				1. Nausea (severity), Vomiting (incidence)	1: Severity of nausea day 1: A < B sign. (*p* = 0.040) Incidence of vomiting: no sign. differences
				2. QoL (impact of CINV on patients’ Health-related QoL)	2: Health-related QoL total score: A > B sign. (*p* < 0.001) Subcategories: A > B sign. in terms of role functioning (*p* = 0.001), fatigue (*p* = 0.002), nausea and vomiting (*p* < 0.001), pain (*p* = 0.017), loss of appetite (*p* < 0.001), constipation (*p* = 0.046). The domains role functioning and loss of appetite reached a minimal clinically sign. change (10 points from baseline levels).
				Salihah:	
				3. Nutrition (dietary intake)	3: Mean energy intake: sign. main effect time and group; p’s < 0.001, no sign. interaction group x time (*p* = 0.638); day 3, 5: A > B sign. (*p* = 0.015; *p* = 0.002) Energy needs met: sign. main effect time and group; p’s < 0.001, no sign. interaction group x time (*p* = 0.604); day 3, 5: A > B sign. (*p* = 0.010; *p* = 0.002) Ratios of carbohydrate, protein, fat, fiber: no sign. differences (*p* = 0.111–0.924).
				4. General perception of aromatherapy administration	4: No sign. differences
				Lua + Salihah:	
				5. Adverse events	5: Lua: very mild dizziness or light-headedness (A: 1) Salihah: not reported
				6. Success of blinding	6: Lua: 93.3% effectiveness Salihah: not reported
Manusirivithaya et al. (2004) [37]	*N* = 48 A = 25 B = 23	Cth: cisplatin (75 mg/m²) Duration: 2 cycles	A: ginger capsules day 1: 0.5 g day 2–5: 1 g/d B: placebo	1. Nausea, vomiting (acute: ≤ 24 h)	1: No sign. differences (nausea: *p* = 0.875; vomiting: *p* = 0.754)
				2. Nausea, vomiting (delayed: day 2–5)	2: No sign. differences (nausea: *p* = 0.294–0.865; vomiting: *p* = 1.000)
				3. Adverse events	3: No sign. differences (*p* = 0.109–1.000) Reported adverse events: restlessness, diarrhea, constipation, headache, dizziness, heartburn, palpitations, akathisia, acute dystonic reaction
				4. Preference for one or the other antiemetic regimen	4: No preference: 41.9%; A: 39.5%; B: 18.6% (*p* < 0.001)
Marx et al. (2017) [38]	*N* = 51 A = 24 B = 27	Cth: moderately or highly-emetogenic Cth Duration: 3 cycles	A: ginger capsules 1.2 g/d B: placebo	1. QoL (CINV-related)	1: Cycle 1: A > B sign., clinical relevance minimal. Nausea-related QoL: A > B sign. (*p* = 0.029) Total CINV-related QoL: A > B sign. (*p* = 0.043) Cycle 2, 3 and for vomiting-related QoL: no sign. differences (*p* > 0.05)
				2. CINV-Symptoms: nausea (acute, delayed), vomiting, retching	2: No sign. differences in prevalence and severity of CINV in all cycles (*p* > 0.05). Subgroup analysis: no sign. difference between participants with and without aprepitant (*p* > 0.05)
				3. Nutrition (nutritional status)	3: No sign. differences in all cycles (*p* > 0.05)
				4. QoL (global cancer-related)	4: A > B sign., clinically relevant: cycle 1 (*p* = 0.015); cycle 3 (*p* = 0.040) No sign. differences in cycle 2 (*p* = 0.077)
				5. Fatigue (cancer-related)	5: A > B sign., clinically relevant: cycle 1 (*p* = 0.006); cycle 3 (*p* = 0.013) No sign. differences in cycle 2 (*p* = 0.23)
				6. CINV-prognostic factors (prevalence): Age, gender, anticipatory CINV, Cth emetogenicity	6: No sign. associations (*p* > 0.05)
				7. Adherence	7: Min. 3 capsules/day: 71% A: 67%, B: 74%
				8. Quality of patient blinding	8: Assignment correctly guessed A vs. B: 63% vs. 30% reasons: taste of ginger (13%), smell of capsules (20%), lack of nausea (60%)
				9. Adverse events	9: Sign. adverse events A vs. B: 1 vs. 3 participants, none assigned to intervention
Montazeri et al. (2013) [39]	*N* = 44 A = 22 B = 22	Cth: cisplatin (50–100 mg) Duration: 2 cycles	A: ginger capsules 1 g/d B: placebo	1. Nausea (frequency, severity)	1: Severity of nausea: results are not presented in an understandable way. 24 h after Cth: severity: A < B sign. (*p* = 0.01) Frequency: no sign. differences
				2. Vomiting / retching (frequency, severity)	2: Severity of vomiting / retching: results are not presented in an understandable way. Frequency of vomiting / retching: no sign. differences
Panahi et al. (2012) [40]	*N* = 100 A = 50 B = 50	Cth: docetaxel, epirubicin, cyclophosphamide Duration: 1 cycle	A: ginger capsules 1.5 g/d B: no additional intervention	1. Nausea, vomiting, retching (prevalence)	1: Prevalence of nausea: 6–24 h after Cth: A < B sign. (*p* = 0.04) Prevalence of vomiting, retching: no sign. differences (*p* = 0.26–0.80)
				2. Nausea, vomiting, retching (severity)	2: No sign. differences (*p* = 0.31–0.98)
				3. Nausea, vomiting, retching (proportion of participants with no / mild / moderate / severe symptoms)	3: No sign. differences (*p* = 0.26–1.00)
				4. Adverse events	4: A: heartburn, headache, vertigo
Ryan et al. (2012) [41]	*N* = 744 A = 187 B = 187 C = 183 D = 188	Cth: not reported Duration: 3 cycles (first cycle: baseline)	Ginger capsules (250 mg) A: 1.5 g/d B: 1 g/d C: 0.5 g/d D: placebo	1. Nausea (severity: acute (mean, maximum))	1: Mean acute nausea: A-C < D sign. (*p* = 0.013); sign. single difference D > C (*p* = 0.046), no sign. differences for other individual comparisons (D vs. B, *p* = 0.076; D vs. A, *p* = 0.738). Maximum acute nausea: A-C < D sign. (*p* = 0.003); sign. single difference D > B (*p* = 0.036), D > C (*p* = 0.017), no sign. differences for D vs. A (*p* = 0.431).
				2. Nausea (delayed)	2: No sign. differences
				3. Nausea (anticipatory)	3: Tests of fixed effects showed that anticipatory nausea (*p* < 0.0001) is a factor in acute CIN
				4. QoL	4: No sign. differences
				5. Vomiting	5: No sign. differences
				collected by the questionnaire, but not defined as an endpoint:	
				6. Adverse events	6: 9x related to intervention (out of 24 total): gastrointestinal symptoms (stage 2 heartburn, bruising/flushing, rash) which led to drop out
Sanaati et al. (2016) [42]	*N* = 65 A = 15 B = 15 C = 15	Cth: single day Cth Duration: 1 cycle	A: ginger capsules 1 g/d B: chamomile C: no additional intervention	1. CINV (frequency, intensity)	1: Intensity of nausea: no sign. differences (*p* = 0.238) Frequency of nausea: sign. effects for A (*p* = 0.013), with sign. difference between A and B (*p* = 0.002) and A and C (*p* = 0.006) Frequency of vomiting: A and B sign. effects (*p* < 0.0001; *p* = 0.02), no sign. group difference between A and B (*p* = 0.177), but sign. differences between A and C (*p* > 0.0001) and B and C (*p* = 0.003)
				collected by the questionnaire, but not defined as an endpoint:	
				2. use of other antiemetics	2–4: not reported
				3. Missed capsules	
				4. Adverse events	
Santos et al. (2023) [43]	*N* = 48 A = 16 B = 16 C = 16	Cth: cisplatin (40 mg/m²) associated with radiotherapy Duration: 6 weeks	A: ginger 2 × 500 mg per day B: ginger 2 × 250 mg per day C: placebo	1. Nausea (number of episodes: acute (24 h after Cth), delayed (day 4); severity)	1: Number of episodes for acute nausea: no sign. differences (*p* = 0.40, *p* = 0.93). Number of episodes for delayed nausea: no sign. change over the 6 weeks (*p* = 0.87) between the 3 groups (*p* = 0.28, *p* = 0.21). Severity: in A and B more participants experienced a lower grade than in C, but no p-value was given.
				2. Vomiting (number of episodes: acute (24 h after Cth), delayed (day 4); severity)	2: Number of episodes for acute and delayed vomiting: no sign. change over the 6 weeks (acute: *p* = 0.35; delayed: *p* = 0.54) between the 3 groups (acute: *p* = 0.85, *p* = 0.54; delayed: *p* = 0.56, *p* = 0.28). Severity: in A and B more participants experienced grade 0 than in C, but no p-value was given.
				3. Symptoms associated with dehydration (incidence of dry mouth (xerostomia), feebleness, thirst, dizziness, dark urine)	3: Results are not presented in an understandable way.
Shokri et al. (2017) [44]	*N* = 49 A = 20 B = 29	Cth: platinum-based adjuvant Cth (carboplatin 5–6 AUC, paclitaxel 175 mg/m²) Duration: 6 cycles	A: ginger capsules 2 g/d B: placebo	1. Poor 12-month outcome (serum CA125 level > 35U, radiologic evidence of metastases, recurrence or death).	1: Serum CA125 level: no sign. differences at all time points (*p* = 0.80). Abnormally elevated CA125 level: no sign. differences (*p* = 0.24). Metastases on CT scan: baseline, 6 months: A < B sign. (*p* = 0.05; *p* = 0.04) No sign. differences at other time points (*p* = 0.18–0.49). Mortality: No sign. differences (*p* = 0.68). Adverse prognoses: A < B sign. (*p* = 0.04)
				2. 12-month disease-free survival	2: No sign. differences (*p* = 0.55)
				3. Side effects of Cth	3: No sign. differences. Side effects: nausea, vomiting (*p* = 0.57), weight loss (*p* = 0.66), peripheral neuropathy (*p* = 0.58), bone marrow depression (*p* = 0.54), transient cortical blindness (*p* = 0.41), other side effects (*p* = 0.11). No intervention-related adverse events.
Shooriabi et al. (2016) [45]	*N* = 40 A = 20 B = 20	Cth: radiotherapy, no Cth Duration: 2 weeks	A: ginger capsules 2 g/d B: placebo	1. Xerostomia symptoms (dry mouth, difficulty chewing/swallowing/speaking, oral burning sensation, bad taste, nightly awakening, grid denture, failure to meet people, non-speaking to people, no leaving the house)	1: A: Sign. improvement: dry mouth (general, daily, nightly), difficulty chewing/swallowing/talking, oral burning sensation, nightly awakening, bad taste, saliva weight (*p* < 0.05). B: Sign. improvement: dry mouth, difficulty chewing / swallowing, oral burning sensation (general, nightly), meeting people, saliva weight (*p* < 0.05).
				2. Saliva quantity (weight, unstimulated)	2: Increase: A > B sign. (*p* < 0.01)
				3. Satisfaction with intervention (improvement in xerostomia, reduction in bad taste, removing of nightly xerostomia, removing nightly awakenings, medication use, improving stuck denture, quality of life, desire to meet people, leave home, higher association to people, ease of using medication, impact on xerostomia, feeling better)	3: A: Improvement in medication use: yes > no (*p* = 0.041) Positive impact on stuck denture: yes > no (*p* = 0.041) B: Positive influence on stuck denture: yes > no (*p* = 0.003) Increased desire to leave the house: yes > no (*p* = 0.041) Ease of using medication/feeling better (unclear indicated which of the two factors the reported outcome refers to): yes < no (*p* = 0.041) Authors indicate that in terms of tend to use medication and positive impact on stuck denture, A > B sign.
Sontakke et al. (2003) [46]	*N* = 60 A = 20 B = 20 C = 20	Cth: cyclophosphamide (500–1000 mg) Duration: 3 cycles	A: ginger capsules 2 g/d B: metoclopramid C: ondansetron	1. Nausea (none = complete control; mild-moderate = partial control; severe = no control)	1: Complete control: A < C sign. (*p* < 0.01), no sign. difference between A and B. partial / no control: no sign. differences
				2. Vomiting (incidence: no vomiting = complete control; 1–2 episodes = partial control; ≥ 3 episodes = no control)	2: Complete control: A < C sign. (*p* < 0.01), no sign. difference between A and B. Partial / no control: no sign. differences. Subgroup analysis: complete control of vomiting: patients with low dose Cth + cytotoxics causing mild emesis: no sign. differences. Patients with low dose Cth + cytotoxics causing severe emesis: A < C sign. (*p* < 0.01), no sign. difference between A and B. Patients with high dose Cth + cytotoxics: A < C sign. higher (*p* < 0.01), no sign. difference between A and B. In all three groups, the incidence of vomiting was sign. higher after 12–24 h than after 0–12 h (*p* < 0.05).
				3. Adverse events	3: Oral ulcers (3), alopecia (5), diarrhea (2). No group assignment or comparison reported. No adverse effects attributable to ginger
Thamlikitkul et al. (2017) [47]	*N* = 34 A = 19 B = 15	Cth: adriamycin-cyclophosphamide regimen Duration: 2 cycles	A: ginger capsules 1 g/d B: placebo	1. Nausea (reduction in score)	1: No sign. differences (acute: *p* = 0.64; delayed: *p* = 0.21; during the first five days of Cth: *p* = 0.30)
				2. Vomiting (incidence)	2: No sign. differences (*p* = 0.5)
				3. Nausea (rate of rescue medication use)	3: No sign. differences (*p* = 0.4)
				4. Incidence of Cth dose reduction or delay	4: Dose reduction: A vs. B: 3% vs. 3% Delay: A vs. B: 9% vs. 6%
				5. Adverse events	5: No sign. difference: fever, fatigue, mucositis, diarrhea, constipation, neutropenia, thrombocytopenia
Uthaipaisanwong et al. (2020) [48]	*N* = 48 A = 24 B = 24	Cth: carboplatin-paclitaxel Duration: 2 cycles	A: ginger capsules 2 g/d B: placebo	1. Nausea (score: mean)	1: Day 1 (acute): A < B sign. (*p* = 0.03) Day 2–5 (delayed): no sign. difference (*p* = 0.39 − 0.94)
				2. Vomiting (frequency)	2: No sign. difference (acute: *p* = 0.78; delayed: *p* > 0.05)
				3. Nausea grade	3: No sign. difference (*p* > 0.05)
				4. Adverse events	4: No sign. difference: diarrhea (*p* = 0.34), constipation (*p* = 0.09), heartburn (*p* = 0.38)
Yekta et al. (2012) [49]	*N* = 98 A = 49 B = 49	Cth: single day Cth Duration: 1 cycle	A: ginger capsules 1 g/d B: placebo	1. Vomiting (anticipatory: 72 h before Cth, acute: up to 24 h after Cth, delayed: 24–72 h after Cth total)	1: A < B sign. (anticipatory: *p* = 0.04; acute: *p* = 0.04; delayed: *p* = 0.003; total sum of 6 days: *p* = 0.002)
				2. Adverse events (anticipatory, acute, delayed)	2: No sign. differences: anticipatory (*p* = 0.2), acute (*p* = 0.06), delayed (*p* = 0.5). Reported adverse event: heartburn
Zick et al. (2009) [50]	*N* = 162 A = 52 B = 53 C = 57	Cth: various Cths Duration: 1 cycle	Ginger capsules A: 2 g/d B: 1 g/d C: placebo	1. CINV (delayed (> 24 h after Cth): prevalence, severity)	1: No sign. difference: prevalence of delayed vomiting (*p* = 0.35 respectively *p* = 0.07), severity of delayed vomiting (*p* = 0.88 respectively *p* = 0.77), prevalence of delayed nausea (*p* = 0.15 respectively *p* = 0.16), severity of delayed nausea regarding patients without the use of aprepitant (*p* = 0.69). Regarding patients who used aprepitant: A and B > C sign. (*p* = 0.01). Single tests: A > B and C sign. (*p* = 0.03)
				2. CINV (acute (≤ 24 h after Cth): prevalence, severity)	2: No sign. difference: prevalence of acute vomiting (*p* = 0.35 respectively *p* = 0.47), severity of acute vomiting (*p* = 0.61 respectively *p* = 0.91), prevalence of acute nausea (*p* = 0.76 respectively *p* = 0.68), severity of acute nausea (*p* = 0.47 respectively *p* = 0.55)
				3. Adverse events / safety	3: No sign. differences: adverse events in total, non-serious adverse events, serious adverse events (*p* = 0.07), dyspnea, gastrointestinal events, laboratory abnormalities (*p* = 0.06). C > A and B sign.: fatigue (*p* = 0.03), miscellaneous adverse events (*p* = 0.02)
				4. Assessment of blinding	4: Investigators could not guess allocation (*p* = 0.27), patients could guess allocation sign. correctly (*p* = 0.01). Reasons: taste of ginger (A: 33%; B: 25%, C: 9%; *p* = 0.01), effect of the capsule (A: 33%, B: 51%, C: 16%; *p* = 0.12)

*Sign.* significant, *Cth* chemotherapy, *CINV* chemotherapy induced nausea and vomiting, *CIN* chemotherapy induced nausea, *QoL* quality of life, *PG-SGA* Patient-Generated Subjective Global Assessment

### Characteristics of included studies

**Fig. 2:**
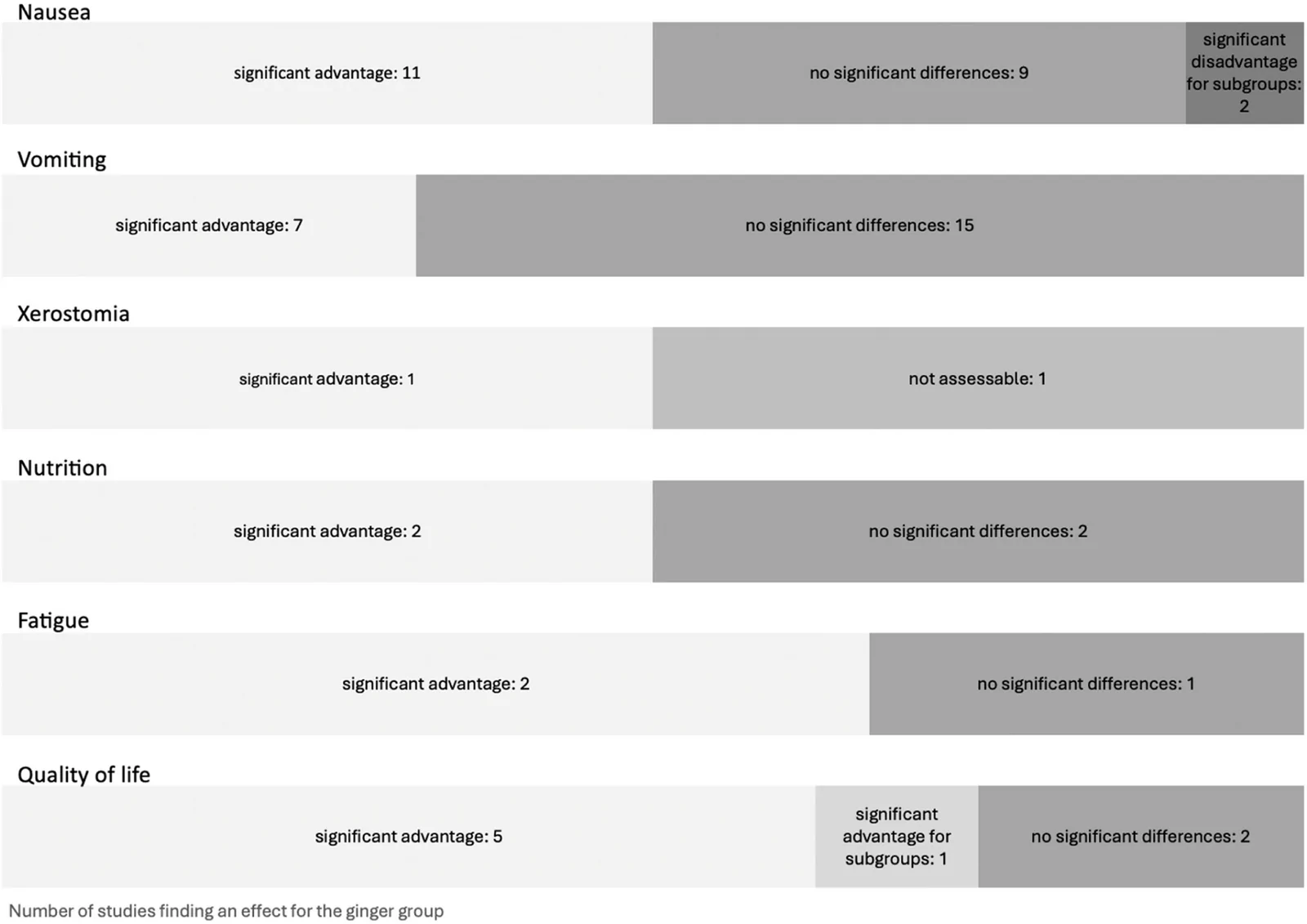
Number of studies finding an effect for ginger

Most of the studies included patients undergoing chemotherapy [25–30, 32–42, 44, 46–50], one study included patients undergoing radiotherapy [45] and one patients undergoing chemotherapy associated with radiotherapy [43]. Most of the chemotherapeutic agents reported were platinum-based [29, 30, 33, 35–37, 39, 43, 44, 48], anthracycline-based [25–28, 35, 40, 47], cyclophosphamide [25–28, 40, 46, 47] or taxane [25–27, 40, 44]. In the studies in which dosages were reported, these ranged between 40 and 103.47 mg/m² for cisplatin [29, 33, 37, 39, 43], between 50 and 60 mg/m² for doxorubicin [27], between 500 and 1000 mg/m² for cyclophosphamide [27, 46] and were 75 mg/m² for docetaxel [27]. Seven studies did not report specific details regarding the chemotherapeutic agents the patients received [32, 34, 38, 41, 42, 49, 50]. Patients with various cancer entities were included in the studies. The most frequently represented types were breast, lung, head and neck, and ovarian cancer. The age of patients ranged approximately from 19 to 88 years, but since some studies only report mean values, no exact statement can be made here. The mean values reported in the studies for the individual groups ranged between 41.8 and 59.5 years, without considerable differences between the groups within a study. The number of randomised participants per group ranged between 15 and 188 across the studies. The distribution of participants across groups was even within the studies [25–30, 32–50]. As intervention most studies used ginger-capsules (0.16–2 g/day) [27, 29, 30, 32, 33, 36–50], one study used capsules with 6-gingerol (0.02 g/day) [35], one powdered ginger (1 g/day) [28], one ginger tea (1.5 g/day) [34] and one used ginger essential oil as aromatherapy (2 drops) [25, 26]. These endpoints were reported: CINV-symptoms [25, 27–30, 32–44, 46–50], xerostomia [43, 45], nutrition [26, 32, 35, 38], fatigue [29, 32, 38], QoL [25, 29, 30, 32, 35, 38, 41]. Figure 2 provides an overview of the number of studies finding a positive effect for ginger on the respective endpoints.

### Excluded studies

Excluded were four randomized controlled trials (RCTs) due to false study type and for one study, only the abstract was available. A list of excluded studies can be seen in the additional file 1 (Table e3).

### Risk of bias in included studies

**Fig. 3:**
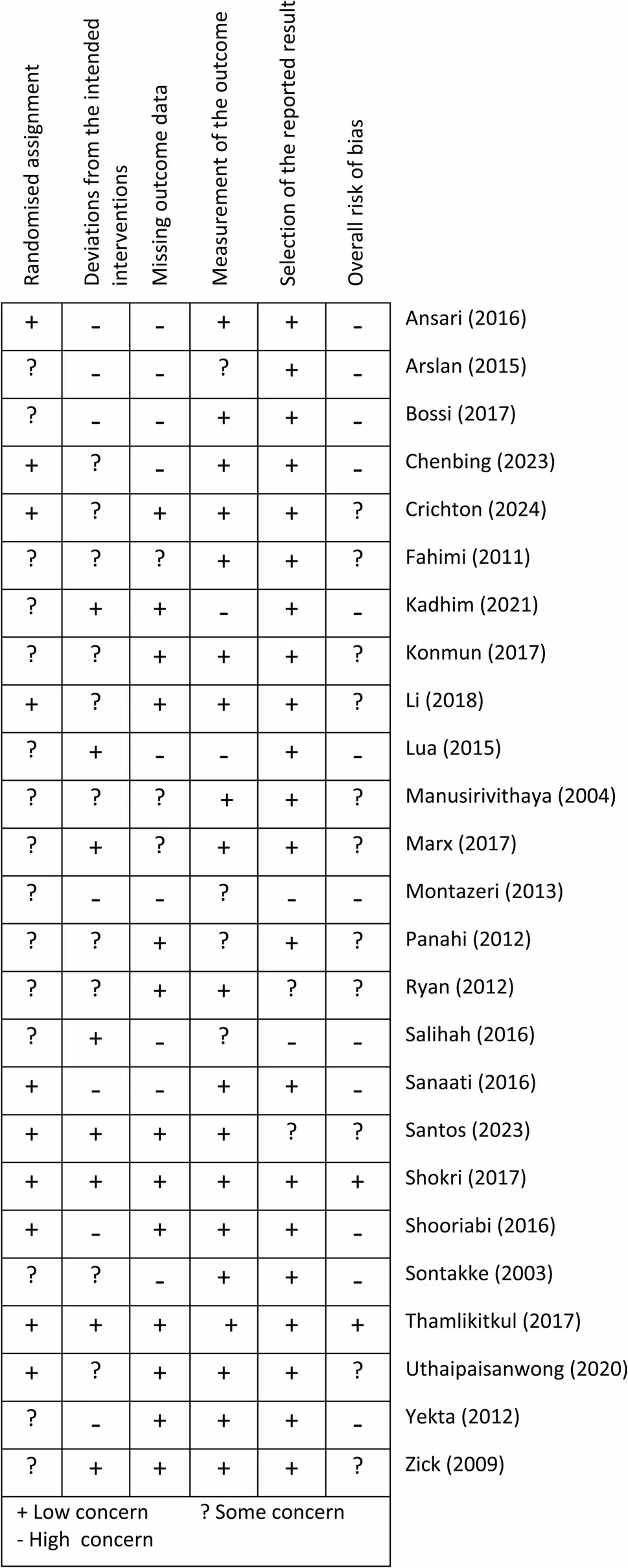
Risk of bias

The results of the RoB analysis are presented in Fig. 3: Risk of Bias (detailed evaluation in the additional file 1, Table e4). Of the 25 studies analysed, twelve show a high RoB [25–30, 34, 39, 42, 45, 46, 49] and eleven show some concerns [32, 33, 35–38, 40, 41, 43, 48, 50], leaving only two studies with a low RoB [44, 47].

### Efficacy of ginger therapy on side effects of cancer treatment

#### CINV-symptoms

Twenty-three studies investigated CINV symptoms like nausea, vomiting, and retching in patients with breast- [25–28, 32, 34, 35, 38, 40–42, 47, 49], gynecological- [32, 34, 35, 37, 43, 44, 48], lung- [29, 32–34, 36, 41] or several other cancers [29, 30, 32–35, 38, 41, 45]. Several studies divided these symptoms in an acute and delayed phase, with mostly the first 24 h after chemotherapy considered acute and the delayed phase from 24 h up to seven days [25, 28–30, 32–41, 43, 47–50]. Modified versions of various questionnaires were used in the majority of studies [25, 27–30, 32–43, 46–50]. The Morrow Assessment of Nausea and Emesis questionnaire [51] or the Rhodes Index of Nausea, Vomiting, and Retching [52] were the most frequently adopted questionnaires [29, 30, 33, 37, 38, 40, 42, 46, 49, 50]. Both questionnaires assess the frequency, severity, and duration of CINV using 17 [51], respectively eight domains [52] with a five-point likert scale. The other questionnaires utilized also use a likert scale [32, 34, 36, 41], visual analogue scale [25, 28, 35, 47] or a categorization into 3 degrees of severity [27, 39, 43, 48]. Most of the studies provided participants with standard antiemetic therapy in addition to the intervention with ginger; only in the study by Sontakke et al. [46] the participants in the ginger group did not receive additional antiemetics.

#### Nausea

Nausea was investigated by 22 studies [25, 27–30, 32–44, 46–48, 50]. A significant positive effect of ginger was found in eleven studies [25, 28, 30, 32, 34, 35, 39–42, 48], from which seven used a placebo as comparison [25, 30, 32, 35, 39, 41, 48]. Of these seven studies, in four the beneficial effect of ginger occurred in the acute phase [25, 39, 41, 48].

Lua et al. [25] found a significant lower severity of nausea on day one for aromatherapy containing ginger essential oil compared to ginger fragrance oil (*p* = 0.040). Ryan et al. [41] included three different dosages of ginger-capsules (0.5 g, 1 g, 1.5 g per day) and found a significant positive effect in the ginger groups compared to placebo concerning mean and maximum acute nausea (mean: *p* = 0.013; maximum: *p* = 0.036). A significant single difference was found for 0.5 g/day ginger group compared to placebo for mean acute nausea and 0.5 g/day (*p* = 0.017) and 1.0 g/day (*p* = 0.036) compared to placebo for maximum acute nausea, but not for 1.5 g/day (*p* = 0.431). Also in Uthaipaisanwong et al. [48] a significant lower severity of nausea in the ginger group was found for acute nausea (*p* = 0.03) and in Montazeri et al. [39] for 24 h after chemotherapy (*p* = 0.01) compared to placebo. About the remaining period of the study by Montazeri et al. [39] no statement can be made, as the results are not presented in a comprehensive way. Konmun et al. [35] generally reported significantly lower severity of nausea for the five day study in the 6-gingerol group compared to placebo (*p* < 0.001). Chenbing et al. [30] found a positive effect of ginger compared to placebo for delayed nausea (*p* = 0.000). Only the group with combined intervention of acupressure and ginger experienced even lower acute (*p* = 0.037) and delayed nausea (*p* = 0.001) than the ginger group. For the severity of delayed nausea, a significant main effect for group (*p* = 0.003) and a significant lower score in the second cycle (*p* = 0.002) was found for ginger compared to placebo in Crichton et al. [32]. Also concerning the incidence of delayed nausea, a significant positive effect of ginger could be found for the third cycle (*p* = 0.002). The differences in the incidence were considered clinically meaningful for cycles two and three and for all cycles concerning the severity of delayed nausea [32].

Of the remaining four studies that did not use a placebo group as a comparison, three studies compared the ginger group with a control group without intervention [28, 34, 40]. A significant effect for lower severity of nausea in the ginger group for day two to five and acute, delayed nausea (*p* < 0.001) in total was found in Arslan et al. [28], for acute (*p* = 0.007) and delayed (*p* = 0.013) nausea in Kadhim et al. [34] and a significant lower prevalence of nausea in the ginger group for 6–24 h after chemotherapy (*p* = 0.04) in Panahi et al. [40].

Another study [42] found a significant effect compared to chamomile (*p* = 0.002) and no intervention (*p* = 0.006) for frequency of nausea, but a false t-test was used with no indication of direction of effect.

No significant positive effect of ginger was found in eleven studies [27, 29, 33, 36–38, 43, 44, 46, 47, 50], from which ten used a placebo group as comparison [27, 29, 33, 36–38, 43, 44, 47, 50]. Eight of these studies could not find a significant difference between ginger and placebo [27, 33, 36–38, 43, 44, 47]. No statistically significant differences were found in Shokri et al. [44] (*p* = 0.57), for acute (*p* = 0.875) and delayed (*p* = 0.294–0.865) nausea in Manusirivithaya et al. [37], as well as in Li et al. [36] for incidence (acute: *p* = 0.174; delayed: *p* = 0.214) and mean score (acute: *p* = 0.246; delayed: *p* = 0.347) and in Fahimi et al. [33] for prevalence (acute: *p* = 0.388; delayed: *p* < 0.999), duration (acute: *p* = 0.93; delayed: *p* = 0.59–0.82), and severity (acute: *p* = 0.14; delayed: *p* = 0.73). In Thamlikitkul et al. [47] no significant difference was found in the acute (*p* = 0.64) and delayed (*p* = 0.21) phase of nausea, as well as over the first five days of chemotherapy (*p* = 0.30), in Ansari et al. [27] across all study cycles in terms of severity (no p-value given) and Marx et al. [38] in terms of prevalence and severity of nausea (*p* > 0.05). In the study by Santos et al. [43] no significant differences in the number of episodes were found for acute (*p* = 0.40, *p* = 0.93) and delayed nausea (*p* = 0.28, *p* = 0.21). Concerning the severity of nausea more participants of both ginger groups (1 g/day and 0,5 g/day) experienced a lower grade, but it was not reported whether this difference was significant.

The other two studies, which did not find a significant positive effect for ginger, even discovered a significant disadvantage for certain subgroups [29, 50]. In Bossi et al. [29] a subgroup analysis with lung cancer patients showed a significant higher incidence of delayed nausea (*p* = 0.042) and nausea between the cycles (intercycle) in the ginger group compared to placebo. For men in the ginger group, the delayed nausea in combination with a visual analogue scale-score of > 25 mm and intercycle nausea was significantly higher than in the placebo group (*p* < 0.05). In Zick et al. [50] a subgroup of patients who used the antiemetic aprepitant showed a significant higher severity of delayed nausea in combination with ginger than placebo (*p* = 0.01). Also the group with 2 g/day experienced significantly higher severity than 1 g/day or placebo (*p* = 0.03).

Sontakke et al. [46] compared the ginger group with the antiemetics ondansetron and metoclopramide. While ginger was significantly inferior to ondansetron (*p* < 0.01), there was no significant difference to metoclopramide concerning complete control of nausea.

#### Vomiting/retching

Vomiting/retching was investigated by 22 studies [25, 27, 28, 30, 32–44, 46–50]. A significant positive effect of ginger was found in seven studies [27, 28, 30, 32, 35, 42, 49], from which five used a placebo as comparison [27, 30, 32, 35, 49]. Ansari et al. [27] found no significant effect in the whole sample, but in the subgroup of patients with chemotherapy-regime doxorubicin + cyclophosphamide the ginger group had significantly less vomiting than the placebo group (*p* < 0.05). Konmun et al. [35] found significantly less vomiting grade three and all grade vomiting in the 6-gingerol group vs. placebo (*p* < 0.001). The 6-gingerol group was significantly superior concerning the complete response rate (no emesis) in cycle one for delayed phase and overall (*p* = 0.004; *p* = 0.001) and over all cycles of the study for vomiting in acute-, delayed phase and overall (*p* = 0.003; *p* < 0.001; *p* < 0.001). A significant effect for ginger was found concerning cases of vomiting for anticipatory (*p* = 0.04), acute (*p* = 0.04), and delayed (*p* = 0.003) phases and the total sum of six days (*p* = 0.002) compared to placebo in Yekta et al. [49] and the number of vomiting on day two, three, five and for the five day mean score (*p* = 0.01; *p* = 0.01; *p* = 0.04; *p* = 0.011) compared to no intervention in Arslan et al. [28]. No significant difference was found for retching [28]. Chenbing et al. [30] found a positive effect of ginger compared to placebo for delayed retching (*p* = 0.001). The acupressure group, as well as the group with combined intervention of acupressure and ginger experienced significant lower acute (*p* = 0.013, *p* = 0.000) and delayed retching (*p* = 0.005, *p* = 0.000) than the ginger group. No significant differences were found for acute and delayed vomiting (*p* = 0.108–0.740) [30]. For delayed vomiting, a significant lower number of episodes was found for ginger compared to placebo for the second and third cycles in Crichton et al. [32] considering it clinically meaningful. Also a main effect for group (*p* = 0.003), time (*p* < 0.001) and interaction effect between group and time (*p* < 0.001) concerning the number of episodes was found for delayed vomiting [32]. Sanaati et al. [42] found a significant effect for the ginger group compared to no intervention (*p* > 0.0001) concerning frequency of vomiting, but a false t-test was used with no indication of direction of effect.

No significant effect of ginger on vomiting/retching was found in 15 studies [25, 33, 34, 36–41, 43, 44, 46–48, 50]. Four studies found no significant group difference concerning vomiting in general [34, 37, 41, 44] and three concerning the frequency of vomiting [36, 39, 48]. Further four studies found no significant group differences concerning the severity [33, 38, 40, 50] and eight studies regarding the incidence of vomiting [25, 33, 36, 38, 40, 43, 47, 50]. In Sontakke et al. [46] ginger was significantly inferior to ondansetron (*p* < 0.01), while there was no significant difference to metoclopramide concerning complete control of vomiting. In the study by Santos et al. [43] no significant differences in the number of episodes were found for acute (*p* = 0.85, *p* = 0.54) and delayed vomiting (*p* = 0.56, *p* = 0.28). Concerning the severity of vomiting it was reported, that more participants of both ginger groups (1 g/day and 0,5 g/day) experienced grad zero than in the placebo group, but it was not reported whether this difference was significant [43]. Montazeri et al. [39] investigated the effect of ginger on the severity of vomiting/retching, but no statement can be made as the results are not presented in a comprehensive way.

No study found any significant disadvantage of ginger in regard to vomiting [25, 27, 28, 33–42, 44, 46–50].

Most of the studies investigating CINV-Symptoms have some concerns [32, 33, 35–38, 40, 41, 43, 48, 50] or a high RoB [25, 27–30, 34, 39, 42, 46, 49]. Only two studies have a low RoB, in which no significant effect of ginger could be found [44, 47]. The studies show several methodological shortcomings (detailed evaluation in the additional file 1, Table e4).

#### Xerostomia

Shooriabi et al. [45] investigated the effect of ginger on patients with head-neck cancer with radiotherapy-induced xerostomia over a study period of two weeks. The ginger group is significant superior compared to placebo in terms of tend to use medication, positive impact on stuck denture (*p* < 0.05), and the increase of saliva quantity (*p* < 0.01). Symptoms associated with dehydration in general were assessed by Santos et al. [43], but no statement can be made as the results are not presented in an understandable way due to only graphical presentation.

The studies investigating the effect of ginger on xerostomia show some concerns [43] and a high RoB [45]. The study by Shooriabi et al. [45] demonstrates a low reporting quality due to inaccurate reporting of the investigated symptoms and missing information on several baseline characteristics.

#### Nutrition

Four studies investigated the effect of ginger on nutrition of patients with breast- or different types of cancer [26, 32, 35, 38] with two of them [32, 38] using the Patient-generated Subjective Global Assessment tool [53]. The tool measures the degree of malnutrition both continuously and as a global rating in three levels (well- / moderately- / malnourished) [53]. The remaining two studies used other parameters [26, 35]. Salihah et al. [26] found significant main effects for time and group (p’s < 0.001) regarding mean energy intake and energy needs met and a higher score on day three (*p* = 0.015; *p* = 0.010) and five (*p* = 0.002; *p* = 0.002) for ginger essential oil. In Marx et al. [38] no significant differences in nutrition status over all cycles (*p* > 0.05) were found between the groups. Also no significant differences were found in Crichton et al. [32] regarding malnutrition (*p* > 0.003), but a lower incidence of malnutrition in the ginger group in the third cycle was considered clinically meaningful. Konmun et al. [35] found a significant advantage for the 6-gingerol group compared to placebo on day 22, 43 and 64 for the appetite score (*p* = 0.001).

Of the four studies investigating nutrition, one has a high RoB [26], three raise some concerns [32, 35, 38] and no study has a low RoB. The studies show several methodological shortcomings (detailed evaluation in the additional file 1, Table e4).

#### Fatigue

Three studies investigated the effect of ginger on the secondary endpoint of fatigue in patients with different types of cancer [29, 32, 38]. Two of the studies [32, 38] used the Functional Assessment of Chronic Illness Therapy Measurement System [54], in which self-reported fatigue is assessed using 13 five-point likert scales. The third study [29] used the Brief Fatigue Inventory questionnaire [55], which categorizes fatigue into three levels of severity on a scale of 1–10. Bossi et al. [29] found no significant differences between ginger and placebo after six days during two cycles. Marx et al. [38] found a significant improvement of fatigue in the ginger group compared to placebo in cycles one and three (*p* = 0.006; *p* = 0.013). Also the study by Crichton et al. [32] found a significant main effect for group (*p* = 0.002), time (*p* = 0.001) and interaction effect between group and time (*p* < 0.001), as well as an advantage of ginger for the first cycle (*p* < 0.001), considering it clinically meaningful.

Of the three studies investigating fatigue one shows a high RoB [29] and two some concerns [32, 38]. The studies show several methodological shortcomings (detailed evaluation in the additional file 1, Table e4).

#### Quality of life

Eight studies investigated endpoints concerning QoL of patients with breast-, lung-, ovarian-, and different cancers [25, 29, 30, 32, 35, 36, 38, 41]. Most frequently the Functional Living Index Emesis score [31] was used [29, 30, 32, 38], which measures the effect of nausea and vomiting on QoL using 18 aspects with a seven-point likert scale [31]. The remaining studies used other questionnaires that also measured different domains using a likert scale, with a higher score corresponding to a higher QoL in each case [25, 35, 36, 41]. Lua et al. [25] found that the ginger essential oil group was significantly superior to ginger fragrance oil for patient‘s health related QoL in total (*p* < 0.001), as well as in the subcategories of role functioning (*p* = 0.001), fatigue (*p* = 0.002), nausea/vomiting (*p* < 0.001), pain (*p* = 0.017), loss of appetite (*p* < 0.001) and constipation (*p* = 0.046). The effect for role functioning and loss of appetite was clinically relevant. Health related QoL was also investigated by Konmun et al. [35], finding a significant improvement for the 6-gingerol group vs. placebo for the total score and for physical- and emotional wellbeing on day 64 (*p* = 0.005; *p* < 0.001; *p* = 0.006). Concerning the mean change of health related QoL, the 6-gingerol group also showed significant clinical differences in comparison with the placebo group. No significant differences for health related QoL could be found in Crichton et al. [32] (*p* > 0.003), but for nausea-, vomiting-, and CINV related QoL significant positive effects for ginger could be found: for vomiting related QoL a significant main effect for group (*p* = 0.001), interaction between group and time (*p* < 0.001) and significantly less decline for cycles two and three (*p* = 0.003, *p* < 0.001), for nausea related QoL a significant main effect for group (*p* = 0.003), time (*p* < 0.001) and significantly less decline for cycle two (*p* = 0.002) and for CINV related QoL a significant main effect for group (*p* < 0.001) and significantly less decline for cycles two and three (*p* < 0.001, *p* = 0.001). Concerning nausea- and CINV related QoL the effect was considered clinically meaningful for all cycles [32]. Marx et al. [38] found significant positive effects of ginger compared to placebo for global cancer related QoL (cycle one: *p* = 0.015; cycle three: *p* = 0.040), nausea-related QoL (cycle one: *p* = 0.029), and total CINV-related QoL (cycle one: *p* = 0.043), but not for vomiting related QoL (*p* > 0.05). Concerning the impact of nausea on daily life, Chenbing et al. [30] found a positive effect for the ginger group compared to placebo for the degree of nausea, activity, cooking, eating, drinking, and personal difficulties (*p* < 0.05). A significant advantage over the ginger group was shown in the acupressure group for degree of nausea / vomiting, activity, eating, drinking, social contact, daily living, and personal difficulties (*p* < 0.05) and in the group with combined intervention of acupressure and ginger for all categories of the impact of nausea and vomiting on daily life (*p* < 0.05) [30]. No significant differences between the groups were found in Li et al. [36] (*p* = 0.884) and Ryan et al. [41] (no p value) investigating QoL in general on day five and four, as well as in Bossi et al. [29] concerning the impact of nausea on daily life on day six of both cycles. But the subgroup analyses for women and patients with head-neck-cancer showed a significant higher score in the ginger group compared to placebo (*p* = 0.048; *p* = 0.038) concerning the ability to maintain daily life [29].

Three studies investigating QoL show a high RoB [25, 29, 30], the remaining five show overall some concerns [32, 35, 36, 38, 41]. No study investigating QoL has a low RoB. The studies show several methodological shortcomings (detailed evaluation in the additional file 1, Table e4).

#### Adverse events

Adverse events of ginger were collected in 18 studies [25, 27–30, 32, 35–38, 40, 41, 44, 46–50]. Of these, two studies found no adverse events [27, 28]. In most of the studies, no significant group differences were found for adverse events in general [29, 30, 35–38, 44, 47–50].

Four studies reported adverse events related to ginger [25, 29, 32, 41]: Lua et al. [25] reported very mild dizziness or light-headedness and Crichton et al. [32] a higher prevalence of reflux in the ginger group compared to placebo (A: 18%, B: 2%). In Bossi et al. [29] the ginger group reported significant more treatment related side effects than the placebo group in general (A: 63; B: 35) and specifically regarding gastrointestinal events. Ryan et al. [41] reported nine adverse events related to intervention (gastrointestinal symptoms, such as stage 2 heartburn, bruising/flushing and rash) which led to drop out of affected individuals. No study has identified serious adverse events for the use of ginger.

Adverse events were not collected in five studies [33, 34, 39, 43, 45] and collected but not reported in two studies [26, 42]. Eight studies collected adverse events non-systematically [25, 26, 35–37, 40, 46, 50] and four studies do not report how adverse events were collected [27, 28, 30, 44].

## Discussion

In the 24 studies investigated, there appear to be no relevant differences between the application forms regarding the results of the studies. The studies with oral intake of ginger can be compared well, especially the studies that administered ginger in capsule form are of similar design. In the studies that compared different dosages, it was observed that there was no advantage or even a disadvantage for dosages above 1 g/day [41, 50]. However, this effect cannot be reproduced across the other studies, as positive effects for ginger were also found in studies that chose 1.5 g [34, 40] or 2 g per day [44–46, 48]. In general, no definitive correlation between dosage and outcome can be identified in the studies included in this review. In order to be able to make a reliable statement on the influence of the dosage on the effectiveness of ginger, further studies are necessary. To ensure safe use, a daily dose of 1 g should not be exceeded due to the indications of a possible disadvantage of higher dosages.

It should be noted that the secondary endpoint of mortality was only analysed in one study [44], although the assessment of mortality is considered standard in supportive studies. Future studies should take this into account.

One major problem, consistent through most of the included studies, is unsuccessful or inadequate blinding. In most studies, subjective parameters were investigated [25, 27–30, 32–43, 45–48, 50], requiring reliable blinding to obtain valid results [56]. But only one single blinded study reports successful blinding [25, 26] and none of the double blinded studies. The difficulties in blinding can be attributed to the intense taste or smell of ginger, which the studies did not seem to be able to eliminate even by administering it in the form of capsules. Due to the lack of blinding in the studies, the results should be interpreted with caution, especially for subjective parameters. A better method must be found in future studies to ensure reliable blinding of the participants.

The most common symptom investigated is CINV. More studies showed an effect on acute nausea than on delayed nausea [25, 28, 34, 35, 39–42, 48]. This effect can be explained by the receptors involved in the different phases. In the acute phase of CINV, 5-HT3 receptors in the intestine play a major role, whereas the delayed phase is more NK-1 mediated [57]. Since ginger has a higher antagonistic effect on 5-HT3 receptors, than on NK-1 receptors, the effect is mainly seen in the acute phase [17]. The disadvantage of men and lung cancer patients found in Bossi et al. [29] was explained by the adverse gastrointestinal events of ginger. Therefore, they hypothesize that in subgroups with a lower risk of CINV, and thus presumably lower CINV symptoms due to chemotherapy, the adverse events would be more significant than possible benefits of ginger intake. Since this subgroup analysis was not pre-planned and there was no stratification of the patients by these factors, the results should be interpreted with caution. Regarding the disadvantage in the aprepitant group of the study by Zick et al. [50], it is striking that also in the study of Bossi et al. [29] all patients received an NK-1 receptor antagonist. This effect could be explained by the mechanism of action of ginger and of the standard antiemetics as NK-1 receptor antagonists on CINV. Ginger increases the motility of the gastrointestinal tract and accelerates gastric emptying [17], which can influence the absorption of orally administered drugs. Since aprepitant is administered orally and already has a bioavailability of only about 65% [58] under normal circumstances, the through ginger accelerated passage of the gastrointestinal tract could further reduce oral bioavailability. To find out whether other factors play a role that are independent of enteric absorption, this effect could be tested with an intravenously administered NK-1 receptor antagonist. However, this observed effect is not reliable, as the sample size of the study was inadequate for detecting differences concerning the endpoints with or without aprepitant, as well as the fact that aprepitant was also applied in other studies [27, 28, 36, 42] and a subgroup analysis did not find significant differences between the groups with and without aprepitant [36]. Based on these different results, it is not possible to assess whether ginger has a negative effect on the efficacy of aprepitant or other antiemetics. To determine this, further studies need to be conducted that systematically investigate whether the combined use of ginger and aprepitant has a disadvantage compared to taking it alone.

More studies find a positive effect of ginger in relation to nausea than for vomiting. Nausea as an endpoint is a very subjective parameter, whereas vomiting can be measured objectively. Therefore, the different results for these endpoints suggest that they may be influenced by the poor blinding of the studies. Due to the inability to precisely determine the extent of the impact of insufficient blinding, it can be said that the results for CINV are only reliable to a limited extent, particularly regarding the subjective endpoint of nausea. Further differences in the studies are the inclusion criteria. Most importantly, some studies only included participants who had previously suffered from CINV [28, 30, 39, 41, 42, 46, 47, 49, 50], while other studies did not meet these restrictions [25, 33–37, 40, 48] or even explicitly included only chemotherapy-naive participants [27, 29, 32, 38, 43, 44]. When comparing these studies, more studies with participants with previous CINV symptoms seem to find a positive effect for ginger than studies with participants who had no previous CINV symptoms. This also explains why in three studies only very few participants suffered from relevant CINV symptoms [34, 40, 48]. These differences in inclusion criteria influence the results of the studies, as the different chemotherapeutic agents and possibly previous CINV symptoms are major influencing factors on CINV [59], which limits the comparability of the studies.

Overall, a positive effect for ginger on CINV could be found in some studies. This effect is consistent with the results of previous reviews assessing the influence of ginger on CINV [20–22]. But, as more studies did not find a significant difference for vomiting, a definitive evaluation of the extent of potential benefits of ginger is not possible. Also, the data on this is still limited due to methodological shortcomings in the trials, so more trials with appropriate methodology are needed to provide a stronger evidence base. Additionally, further studies are required to investigate possible disadvantages for certain subgroups to ensure safe use.

Concerning the endpoint of xerostomia, the indication of a positive effect of ginger is limited by methodological shortcomings of the studies. To reliably assess the effect of ginger on xerostomia further high-quality research is necessary.

The studies investigating the effect of ginger on nutrition are difficult to compare, as different factors were measured. Indications of a potential positive influence of ginger on the nutrition of patients support the beneficial effect of additional ginger intake by oncological patients with CINV. However, further research is necessary to conclusively assess the extent of the influence of ginger on the nutrition of oncological patients as the differences were not considered significant in half of the studies and subjective parameters were used.

There were also indications of a positive effect of ginger on fatigue. The validity of the results in this context is also limited by methodological drawbacks and insufficient blinding. So further research with more reliable blinding of the participants is needed.

Five out of eight studies investigating endpoints concerning QoL found a positive effect of ginger for the entire sample [25, 30, 32, 35, 38] and one study for the subgroups of women and patients with head-neck-cancer [29]. The authors explain the positive effect in women and patients with head-neck-cancer by the fact that the risk of CINV is increased in these subgroups and that the positive effect of ginger would therefore be stronger in comparison to possible adverse events [29]. However, since this subgroup analysis was not pre-planned, the results should be interpreted with caution. The demonstrated influence of ginger on QoL is limited by the inadequacy of blinding and methodological drawbacks of the studies, so more research is needed to definitively determine the benefits of additional ginger intake.

Adverse events of ginger were found in four studies identifying mainly mild adverse events [25, 29, 32, 41]. Bruising, which was described as an adverse event in Ryan et al. [41], could indicate affected coagulation. In fact, preclinical studies were able to determine an influence of ginger on coagulation, but this effect could not be verified in clinical studies [60]. Accordingly, it is rather unlikely that this effect plays a relevant role in humans at the doses used in the studies. The dizziness reported by Lua et al. [25] could most likely be linked to ginger via low blood pressure, since preclinical studies found a blood pressure-lowering effect of ginger [60]. However, this is unlikely, as the amount ingested through aromatherapy would probably not be sufficient for such an effect, as the preclinical studies used significantly higher amounts of ginger [60]. The reported adverse gastrointestinal events are most likely to be associated with an irritant effect of ginger on the gastric mucosa. In previous studies a gastric irritant effect of ginger was found for dosages above six grams [60]. In the studies investigated here, lower doses were chosen, but it could be assumed that since oncological patients already have mucosal irritation due to cytotoxic cancer therapies, lower doses of ginger could be high enough to cause symptoms [61]. Beyond these mild adverse events, no study reports severe adverse events of ginger. Overall, ginger intake can be classified as safe, with no serious adverse events to be expected. However, to safely assess the influence of the irritant effect on the mucosa in oncological patients, further research is needed in this area. Similarly, further targeted research is needed to assess a possible interaction between ginger and other antiemetics such as aprepitant, to enable the safe use of ginger.

### Limitations of this work

Some limitations must be mentioned in this systematic review. Concerning the inclusion criteria only studies with adult participants were included, studies with children and adolescents were excluded. Furthermore, only publications in English or German were included. Accordingly, the literature search on ginger in the therapy of oncological patients can be expanded in future research regarding the participants and languages included. Overall, the included studies often described the symptoms only imprecisely due to the heterogeneity of both CINV and cancer entities, making it impossible for us to provide a more precise description in our review.

Moreover, due to the high heterogeneity of the studies and the data, we did not conduct a meta-analysis.

## Conclusion

The proportion of significant outcomes does not allow definitive conclusions regarding the extent of the efficacy of ginger in supportive therapy against side effects of oncological treatment. Nevertheless, a considerable number of studies identified a beneficial impact of ginger on CINV, nutrition, fatigue, and QoL, with only few reported adverse events. This suggests that ginger may be considered a promising candidate for complementing the supportive therapy against side effects of cancer treatment of oncological patients. Serious adverse events are rare, but interaction with aprepitant have to be considered, as well as adverse effects from high dosages.

### Future directions

To reliably assess the influence of ginger in supportive therapy against side effects of cancer treatment and to safely evaluate possible interactions, further research with methodologically high-quality RCTs with detailed reporting is necessary. Therefore, future studies should prioritize reliable blinding, as well as precise specification of inclusion criteria and participant characteristics to ensure a stronger evidence base. Differences in dosage, and especially possible disadvantages of high doses, should be investigated further to ensure safe use. To evaluate potential interactions between ginger and other antiemetic drugs, high-quality RCTs specifically designed to address this issue are needed. For example, by testing antiemetics with different mechanisms of action in combination with ginger, to identify a possible influence on certain mechanisms of action, so that this can be taken into account or avoided in clinical use.

## Supplementary Information

Additional file 1. Online material: Search strategy; Characteristics of included studies; Excluded studies; Risk of Bias table.

## Data Availability

All data generated or analysed during this study are included in this published article and its supplementary information files.

## Abbreviations

CINV: Chemotherapy-induced nausea and vomiting
5-HT3: 5-hydroxytryptamine 3 receptor
NK-1: Neurokinin-1 receptor
QoL: Quality of life
RoB: Risk of bias
RCTs: Randomized controlled trials
