# Supplementary material for: Ginger in supportive therapy against side effects of oncological treatment: a systematic review
Source: BMC Complement Med Ther. 2026 Aug 18;26:249. doi: 10.1186/s12906-026-05530-z (PMC13488015; doi:10.1186/s12906-026-05530-z)
Supplement: Supplementary file 1 [file 12906_2026_5530_MOESM1_ESM.docx]

# Online material

### Table e1: Search strategy

| **Database** | **Search strategy** |
| --- | --- |
| Ovid Medline | **1** GINGER/ or (Ginger or Zingiber officinale* or gingerol).mp.  **2** exp neoplasms/ or neoplasm$.mp or cancer$.mp. or tumo?r$.mp. or malignan$.mp. or oncolog$.mp. or carcinom$.mp. or leuk?emia.mp. or lymphom$.mp. or sarcom$.mp.  **3** 1 AND 2  **4** limit 3 to english or limit 3 to german  **5** (4 and humans/) or (4 not animals/)  **6** ((((comprehensive* or integrative or systematic*) adj3 (bibliographic* or review* or literature)) or (meta-analy* or metaanaly* or "research synthesis" or ((information or data) adj3 synthesis) or (data adj2 extract*))).ti,ab. or (cinahl or (cochrane adj3 trial*) or embase or medline or psyclit or (psycinfo not "psycinfo database") or pubmed or scopus or "sociological abstracts" or "web of science" or central).ab. or ("cochrane database of systematic reviews" or evidence report technology assessment or evidence report technology assessment summary).jn. or Evidence Report: Technology Assessment*.jn. or (network adj1 analy*).ti,ab.) or (((review adj5 (rationale or evidence)).ti,ab. and review.pt.) or meta-analysis as topic/ or Meta-Analysis.pt.)  **7** Randomi?ed controlled trial?.pt. or controlled clinical trial?.pt. or randomi?ed.ti,ab.or placebo.ti,ab. or drug therapy.sh. or randomly.ti,ab. or trial?.ti,ab. or group?.ti,ab.  **8** 5 AND (6 OR 7) |
| Ovid Embase | **1** ginger/ or gingerol/ or (Ginger or Zingiber officinale* or gingerol).mp.  **2** exp neoplasm/ or neoplasm$.mp or cancer$.mp. or tumo?r$.mp. or malignan$.mp. or oncolog$.mp. or carcinom$.mp. or leuk?emia.mp. or lymphom$.mp. or sarcom$.mp.  **3** 1 AND 2  **4** limit 3 to english or limit 3 to german  **5** (4 and humans/) or (4 not animals/)  **6** ((((comprehensive* or integrative or systematic*) adj3 (bibliographic* or review* or literature)) or (meta-analy* or metaanaly* or "research synthesis" or ((information or data) adj3 synthesis) or (data adj2 extract*))).ti,ab. or (cinahl or (cochrane adj3 trial*) or embase or medline or psyclit or (psycinfo not "psycinfo database") or pubmed or scopus or "sociological abstracts" or "web of science" or central).ab. or ("cochrane database of systematic reviews" or evidence report technology assessment or evidence report technology assessment summary).jn. or Evidence Report: Technology Assessment*.jn. or (network adj1 analy*).ti,ab.) or (exp Meta Analysis/ or ((data extraction.ab. or selection criteria.ab.) and review.pt.))  **7** crossover procedure/ or double blind procedure/ or randomized controlled trial/ or single blind procedure/ or (random$ or factorial$ or crossover$ or (cross adj1 over$) or placebo$ or (doubl$ adj1 blind$) or (singl$ adj1 blind$) or assign$ or allocat$ or volunteer$).ti,ab,de.  **8** 5 AND (6 OR 7) |
| Cochrane | **#1** [mh Ginger] or Ginger or Zingiber officinale or gingerol  **#2** [mh neoplasms] or neoplasm* or cancer? or tum*r? or malignan* or oncolog* or carcinom* or leuk*mia or lymphoma? or sarcoma?  **#3 #**1 AND #2 |
| Ebsco - PsychINFO | **S1** TX Ginger or TX Zingiber officinale* or TX gingerol  **S2** ((DE "Neoplasms" OR DE "Benign Neoplasms" OR DE "Breast Neoplasms" OR DE "Endocrine Neoplasms" OR DE "Leukemias" OR DE "Melanoma" OR DE "Metastasis" OR DE "Nervous System Neoplasms" OR DE "Terminal Cancer") OR (TX neoplasm* OR TX cancer OR TX tumo#r OR TX malignan* OR DE „oncology“ OR TX oncolog* OR TX carcinom* OR TX leuk#emia OR TX lymphoma OR TX sarcoma))  **S3** (LA German OR LA English)  **S4** S1 AND S2 AND S3  **S5** ((comprehensive* OR integrative OR systematic*) N3 (bibliographic* OR review* OR literature)) OR (meta-analy* or metaanaly* or "research synthesis" OR ((information OR data) N3 synthesis) OR (data N2 extract*)) OR ((review N5 (rationale OR evidence)) AND DE "Literature Review") OR (AB(cinahl OR (cochrane N3 trial*) OR embase OR medline OR psyclit OR pubmed OR scopus OR "sociological abstracts" OR "web of science" OR central)) OR DE "Meta Analysis" OR (network N1 analy*)  **S6** DE "Treatment Effectiveness Evaluation" OR DE "Treatment Outcomes" OR DE "Psychotherapeutic Outcomes" OR DE "Placebo" or DE "Followup Studies" OR placebo* OR random* OR "comparative stud*" OR (clinical N3 trial*) OR (research N3 design) OR (evaluat* N3 stud*) OR (prospectiv* N3 stud*) OR ((singl* OR doubl* OR trebl* OR tripl*) N3 (blind* OR mask*)  **S7** S4 AND (S5 OR S6) |
| Ebsco- CINAHL | **S1** (MH "Ginger") or TX Ginger or TX Zingiber officinale* or TX gingerol  **S2** (MH "Neoplasms+" OR TX neoplasm* OR TX cancer OR TX tumo#r OR TX malignan* OR TX oncolog* OR TX carcinom* OR TX leuk#emia OR TX lymphoma OR TX sarcoma)  **S3** (LA German OR LA English)  **S4** S1 AND S2 AND S3  **S5** (TI (systematic* n3 review*)) or (AB (systematic* n3 review*)) or (TI (systematic* n3 bibliographic*)) or (AB (systematic* n3 bibliographic*)) or (TI (systematic* n3 literature)) or (AB (systematic* n3 literature)) or (TI (comprehensive* n3 literature)) or (AB (comprehensive* n3 literature)) or (TI (comprehensive* n3 bibliographic*)) or (AB (comprehensive* n3 bibliographic*)) or (TI (integrative n3 review)) or (AB (integrative n3 review)) or (JN “Cochrane Database of Systematic Reviews”) or (TI (information n2 synthesis)) or (TI (data n2 synthesis)) or (AB (information n2 synthesis)) or (AB (data n2 synthesis)) or (TI (data n2 extract*)) or (AB (data n2 extract*)) or (TI (medline or pubmed or psyclit or cinahl or (psycinfo not “psycinfo database”) or “web of science” or scopus or embase)) or (AB (medline or pubmed or psyclit or cinahl or (psycinfo not “psycinfo database”) or “web of science” or scopus or embase or central)) or (MH “Systematic Review”) or (MH “Meta Analysis”) or (TI (meta-analy* or metaanaly*)) or (AB (meta-analy* or metaanaly*)) or network n1 analy*  **S6** (MH "Clinical Trials+") or PT Clinical trial or TX clinic* n1 trial* or TX ( (singl* n1 blind*) or (singl* n1 mask*)) or TX ((doubl* n1 blind*) or (doubl* n1 mask*)) or TX ( (tripl* n1 blind*) or (tripl* n1 mask*)) or TX ((trebl* n1 blind*) or (trebl* n1 mask*)) or TX randomi* control* trial* or (MH "Random Assignment") or TX random* allocat* or TX placebo* or MH "Placebos") or MH "Quantitative Studies") or TX allocat* random*  **S7** S4 AND (S5 OR S6) |

### Table e2: Characteristics of included studies

| **Reference** | **Study type** | **N / Cancer type / Cth / Drop Out** | **Intervention (A) / Control (B-D) / Duration** | **Endpoints** | **Outcomes** |
| --- | --- | --- | --- | --- | --- |
| Ansari (2016) | RCT | N = 150  Cancer type: breast cancer  Cth: doxorubicin based Cth  Antiemetic therapy: dexamethasone, aprepitant, granisetron  Drop out: 31 | A: ginger capsules (250 mg) 2 x 2 per day  B: placebo  Duration: 3 cycles | 1. Nausea (severity)  2. Vomiting (severity)  3. Adherence | **1:** No significant differences between the groups  Subgroup analyses of different Cth-combinations: no significant differences between the groups  **2**: No significant differences between the groups  Subgroup analyses of different Cth-Combinations: Cth-regime doxorubicin + cyclophosphamide: in arm A significant lower severity of vomiting than in arm B: 0.64 ± 0.87 vs. 1.13 ± 1.12 (p < 0.05)  **3:** Arm A vs. arm B: 76 % vs. 82 % |
| Arslan (2015) | RCT | N = 60  Cancer type: breast cancer  Cth: anthracycline based Cth  Antiemetic therapy: palonosetron, dexamethasone, an antihistamine, ranitidine, aprepitant  Drop out: 0 | A: powdered ginger 2 x 500 mg per day  B: no additional intervention  Duration: 2 cycles | 1. Nausea (severity)  2. Vomiting (number of episodes)  3. Retching (number of episodes) | **1:** Day 2 - 5: in arm A significant lower severity of nausea than in arm B: T2: 3.8 ± 1.9 vs. 6.3 ± 1.9 (p < 0.001)  T3: 3.8 ± 1.8 vs. 6.5 ± 1.8 (p = 0.001)  T4: 3.7 ± 1.9 vs. 6.3 ± 1.8 (p = 0.001)  T5: 2.8 ± 1.7 vs. 5.4 ± 2.3 (p = 0.001)  Five day mean score of nausea severity: in arm A significant lower severity than in arm B: 3 ± 1.5 vs. 5.1 ± 1.5 (p < 0.001)  Acute and delayed nausea severity: in arm A significant lower severity than in arm B: acute: 1.6 ± 1.1 vs. 3.9 ± 1.6 (p < 0.001) (n = 36 (A: 15, B: 21)); delayed: 3.6 ± 1.8 vs. 6.1 ± 1.7 (p < 0.001)  **2:** Day 2, 3, 5: in arm A significant lower number of vomiting than in arm B: T2: 0 ± 0.1 vs. 0.3 ± 0.6 (p = 0.01)  T3: 0 ± 0.1 vs. 0.2 ± 0.4 (p = 0.01)  T5: 0 ± 0 vs. 0.2 ± 0.4 (p = 0.04)  Five day mean score of the number of vomiting: in arm A significantly lower than in arm B: 0 ± 0 vs. 0.2 ± 0.3 (p = 0.011)  **3:** No significant differences between the groups |
| Bossi (2017) | RCT | N = 251  Cancer type: solid tumors  Cth: high-dose cisplatin  Antiemetic therapy: NK-1 receptor antagonist, 5-HT3  receptor antagonist, dexamethasone  Drop out: 97 | A: ginger capsules 4 x 40 mg per day  B: placebo  Duration: 2 cycles | 1. Incidence of delayed nausea and of significant delayed nausea  2. Incidence of intercycle nausea and of significant intercycle nausea  3. Incidence of anticipatory nausea and of significant anticipatory nausea  4. Intensity of nausea  5. Impact of nausea on daily life (ability to maintain daily life)  6. Fatigue  7. Adverse events | **1:** Incidence of delayed nausea: in total no significant differences between the groups (cycle 1: p = 0.851, cycle 2: p = 0.379). Subgroup analysis: lung cancer patients in arm A significantly higher than in B: OR 2.67 95 % CI 1.03/6.87; p = 0.042 (cycle 2)  Incidence of significant delayed nausea: in total no significant differences between the groups (cycle 1: p = 0.163, cycle 2: p = 0.239). Subgroup analysis: men in arm A significantly higher than in B: OR 2.74 95 % CI 1.28/5.85; p < 0.05  **2:** Incidence of intercycle nausea: in total no significant differences between the groups (cycle 1: p = 0.367, cycle 2: p = 0.417). Subgroup analysis: lung cancer patients in arm A significantly higher than in B: OR 2.77 95 % CI 1.16/6.64 (in total); OR 4.27 95 % CI 1.60/11.37 (cycle 1)  Incidence of significant intercycle nausea: in total no significant differences between the groups (cycle 1: p = 0.074, cycle 2: p = 0.361). Subgroup analysis: men in arm A significantly higher than in B: OR 2.38 95 % CI 1.04/5.44; p < 0.05  **3:** Incidence of anticipatory nausea: in total no significant differences between the groups (cycle 1: p = 0.823, cycle 2: p = 0.629).  Incidence of significant anticipatory nausea: in total no significant differences between the groups (cycle 1: p = 0.666, cycle 2: p = 0.536).  **4:** No significant differences between the groups (p = 0.414 - 0.959)  **5:** In total no significant differences between the groups. Subgroup analysis: women and head-neck-cancer patients reached in arm A significant higher scores than in B: 7.31; 95 % CI 14.56/0.06, p = 0.048 respectively 7.43; 95 % CI 14.42/0.43, p = 0.038  **6:** No significant differences between the groups  **7:** In total no significant differences between the groups. Regarding treatment related side effects arm A reported significant more side effects than B (A: 63; B: 35). Arm A reported more gastrointestinal events than B. |
| Chenbing (2023) | RCT | N = 172  Cancer type: gastric, rectal, colon, other  Cth: oxaliplatin regimes (150 / 200mg)  Antiemetic therapy: routine treatment and nursing: based on the guidelines of the NCCN, antiemetic, stomach-protecting, anti-aller­gic treatment diet guidance,  Drop out: 12 (3 in each group) | A: powdered ginger 2 x 500 mg per day (30 min before Cth)  B: acupressure of P6 (anterior on the forearm, 3-finger widths up from the wrist between the tendons of the palmaris longus and flexor carpi radialis) (for 10 min (30 min before Cth)  C: joint (acupressure and ginger as mentioned)  D: no additional intervention  Duration: 1 cycle | 1. Nausea (acute (day 1), delayed (day 2-5))  2. Vomiting / retching (acute (day 1), delayed (day 2-5))    3. Functional living index (for nausea and vomiting: degree, activity, cooking, eating, drinking, social contact, daily living, personally difficulties)  4. Benefit finding  5. Treatment satisfaction  6. Adverse events | **1:** Acute: in arm A significantly higher than in arm C: 2.81 ± 2.94 vs. 1.46 ± 2.08 (p = 0.037). No significant differences between arm A and B (p = 0.574) and arm A and D (p = 0.109).  Delayed: in arm A significantly higher than in arm C: 2.69 ± 2.09 vs. 0.93 ± 1.47 (p = 0.001). In arm A significantly lower than in arm D: 2.69 ± 2.09 vs. 4.83 ± 3.01 (p = 0.000). No significant differences between arm A and B (p = 0.142).  **2:** Acute vomiting: no significant differences between arm A and B (p = 0.320), A and C (p = 0.112), A and D (p = 0.740).  Delayed: no significant differences between arm A and B (p = 0.153), A and C (p =0.108), A and D (p = 0.187).  Acute retching: in arm A significantly higher than in arm B and C: 1.71 ± 1.89 vs. 0.83 ± 1.38 (p = 0.013) and vs. 0.43 ± 1.04 (p = 0.000). No significant differences between arm A and D (p = 0.725).  Delayed retching: in arm A significantly higher than in arm B and C: 1.54 ± 1.43 vs. 0.64 ± 1.14 (p = 0.005) and vs. 0.25 ± 0.61 (p = 0.000). In arm A significantly lower than in arm D: 1.54 ± 1.43 vs. 2.58 ± 2.08 (p = 0.001).  **3:** Nausea: in arm A significant higher score than in D concerning the degree of nausea, activity, cooking, eating, drinking, and personal difficulties (p < 0,05). In arm A significantly lower than in B concerning the degree of nausea, activity, cooking, eating, drinking, social contact, daily living and personal difficulties (p < 0.05). In arm A significantly lower than in C concerning all categories (p < 0.05).  Vomiting: No significant difference between A and D (p > 0.05). In arm A significantly lower than in B concerning degree, activity, eating, drinking, social contact, daily living and personal difficulties (p < 0.05). In arm A significantly lower than in C concerning all categories (p < 0.05).  **4:** No significant differences between the groups (p < 0.05).  **5:** No significant differences between arm A and other groups (p < 0.05).  **6:** No significant differences between the groups (p < 0.05). |
| Crichton (2024) | RCT | N = 103  Cancer type: breast (43%), lung (18%), lymphoma (17%), digestive (5%), urogenital (5%), gynecological (5%), other (7%)  Cth: single day moderately to highly emetogenic Cth  Antiemetic therapy: no restrictions on prescribed antiemetics (5-HT3, NK-1, dopamine receptor antagonists, steroids in different combinations)  Drop out: 33 | A: ginger 4 x 300 mg per day (for 5 days per cycle)  B: placebo  Duration: 3 cycles | 1. Chemotherapy-induced nausea related  QoL  2. Vomiting- and CINV-related QoL  3. Health-related QoL  4. Incidence  and severity of CINV (anticipatory: one day before Cth; acute: 12-24h after Cth; delayed: 4 days after Cth)  5. Fatigue  6. Nutrition status  7. Mental health (depression and  anxiety)  8. Adverse events | **1:** Main effect for group (partial η² = 0.09 [medium effect]; p = 0.003) and for time (partial η² = 0.07 [medium effect]; p < 0.001). Arm A significant less decline than in B for cycle 2 (partial η² = 0.09 [medium effect]; p = 0.002). Clinically meaningful for all cycles.  **2:** Vomiting related QoL: main effect for group (partial η² = 0.09 [medium effect]; p = 0.001) and interaction effect between group and time (partial η² = 0.08 [medium effect]; p < 0.001). In arm A significant less decline than in B for cycle 2 (partial η² = 0.08 [medium effect]; p = 0.003) and 3 (partial η² = 0.11 [medium effect]; p < 0.001).  CINV related QoL: main effect for group (partial η² = 0.11 [medium effect]; p < 0.001). In arm A significant less decline than in B for cycle 2 (partial η² = 0.11 [medium effect]; p < 0.001) and 3 (partial η² = 0.10 [medium effect]; p = 0.001). Clinically meaningful for all cycles.  **3:** No significant differences between the groups (p > 0.003).  **4:** Incidence and severity of anticipatory and acute nausea: no significant differences between the groups (p > 0.003).  Incidence of delayed nausea: in arm A significantly lower than in B at cycle 3 (30% lower) (Cramer’s V = 0.7 [large effect]; p = 0.002). Clinically meaningful for cycle 2, 3.  Severity of delayed nausea: main effect for group (partial η² = 0.09 [medium effect]; p = 0.003). Arm A significant superior to arm B in cycle 2 (partial η² = 0.09 [medium effect]; p = 0.002). Clinically meaningful for all cycles.  No anticipatory vomiting reported at any cycle.  Acute vomiting: no significant differences between the groups (p > 0.003).  Delayed vomiting: main effect for group (partial η² = 0.08 [medium effect]; p = 0.003), time (partial η² = 0.22 [large effect]; p < 0.001) and interaction effect between group and time (partial η² = 0.11 [medium effect]; p < 0.001) concerning number of episodes. In arm A significant lower incidence than in B at cycle 2 (23% lower) (Cramer’s V = 0.7 [large effect]; p = 0.001) and at cycle 3 (21% lower) (Cramer’s V = 0.7 [large effect]; p = 0.001), clinically meaningful.  **5:** Main effect for group (partial η² = 0.09 [medium effect]; p = 0.002), time (partial η² = 0.07 [medium effect]; p = 0.001) and interaction effect between group and time (partial η² = 0.12 [medium effect]; p < 0.001). Arm A significant superior to B at cycle 1 (partial η² = 0.18 [large effect]; p < 0.001), clinically meaningful.  **6:** Main effect for time for PG-SGA score (partial η² = 0.11 [medium effect]; p < 0.001). No significant differences between the groups regarding malnutrition (p > 0.003), but lower incidence of malnutrition in arm A than B in cycle 3 was considered clinically meaningful.  **7:** No significant differences between the groups (p > 0.003).  **8:** No serious adverse events related to the intervention. In total 19 mild and 1 moderate adverse event possibly related to the intervention. Reflux was more prevalent in arm A than in arm B (A: 18%, B:2%). |
| Fahimi (2011) | RCT | N = 50  Cancer type: any type of cancer  Cth: cisplatin-based regimens  Antiemetic therapy: 5-HT3 antagonist (granisetron) + corticostreoid (hydrocortisone)  Drop out: 14 | A: ginger capsules (250 mg) 2 x 2 per day  B: placebo  Duration: 2 cycles | 1. Prevalence, severity, and duration of acute (day 1) and delayed (day 2 - 3) nausea  2. Prevalence, severity, and duration of acute (day 1) and delayed (day 2 - 3) vomiting | **1:** No significant differences between the groups regarding the prevalence of acute (p = 0.388) and delayed (p < 0.999) nausea, the severity of acute (p = 0.14) and delayed (p = 0.73) nausea, the duration of acute (p = 0.93) and delayed (day 2: p = 0.59; day 3: p = 0.82) nausea.  **2:** No significant differences between the groups regarding the prevalence of acute (p = 0.070) and delayed (day 2: p = 0.687; day 3: p < 0.999) vomiting, the severity of acute (p = 0.14) and delayed (day 2: p = 0.72; day 3: p = 0.78) vomiting |
| Kadhim (2021) | RCT | N = 60  Cancer type: any type of cancer except hematological malignancies  Cth: not reported  Antiemetic therapy: “standard therapy”  Drop out: 0 | A: ginger tea (1.5 g per day)  B: no additional intervention  Duration: 1 cycle | 1. Acute and delayed vomiting  2. Acute and delayed nausea  3. Severity of acute and delayed nausea | **1:** No significant differences between the groups regarding acute (p = 0.798) and delayed (p = 0.779) vomiting  **2:** No significant differences between the groups regarding acute (p = 0.182) and delayed (p = 0.112) nausea  **3:** In arm A significant lower severity of acute (p = 0.007) and delayed (p = 0.013) nausea than in B. |
| Konmun (2017) | RCT | N = 94  Cancer type: solid tumors  Cth: moderately to highly emetogenic adjuvant Cth  Antiemetic therapy: ondansetron, metoclopramide, dexamethasone  Drop out: 13 | A: 6-gingerol capsules (5 mg) 2 x 2 per day  B: placebo  Duration: 12 weeks (3 cycles) | 1. Complete response rate (no emesis / rescue treatment) for all cycles of Cth (overall, acute (≤ 24 h), delayed (24 – 120 h))  2. Complete response rate for cycle 1  3. Severity of nausea and vomiting  4. Tolerability  5. Appetite score  6. Health-related quality of life (HRQoL)  7. Adverse events  8. Adherence | **1:** Complete response rate: overall, acute- and delayed phase: in arm A significantly higher than in B.  A vs. B: overall: 77 % vs. 32 % (p < 0.001)  Acute phase: 88 % vs. 58 % (p = 0.003)  Delayed phase: 77 % vs. 32 % (p < 0.001)  **2**: Complete response rate for cycle 1: overall, delayed phase: in arm A significantly higher than in B.  A vs. B: overall: 85 % vs. 49 % (p = 0.001)  Delayed phase: 85 % vs. 54 % (p = 0.004)  Acute phase: no significant differences between the groups (p = 0.057)  **3**: All grade, grade 3 vomiting: in arm A significantly lower than in B. A vs. B:  All grade: 22 % vs. 68 % (p < 0.001)  Grade 3: 0 % vs. 19 % (p < 0.001).  No grade 4 vomiting was observed in this study.  Nausea: in arm A significantly lower severity than in B: A vs. B: mild: 55 % vs. 17 %; moderate: 15 % vs. 39 %; severe: 5 % vs. 34 % (p < 0.001)  **4:** None of the patients was discontinued from the study due to toxicity. No dose reduction was required in both groups. 3 patients discontinued the study early: A: 1 patient because of grade 3 vomiting.  B: 2 patients due to termination of Cth after cycle 2 / due to grade 2 dyspepsia.  **5:** In arm A significantly lower appetite-score than in B for day 22, 43, 64, adjusted with baseline values of day 1: r -1.65, 95 %  CI -2.64 to -0.67; p = 0.001  **6:** In arm A significantly improvement in mean change of the total score (p = 0.005), physical (p < 0.001) and emotional (p = 0.006) wellbeing compared to B. In mean change arm A is also clinically different to B.  No significant group differences in social or family wellbeing (p = 0.203) and functional wellbeing (p = 0.147)  **7:** In general no significant differences between the groups (p = 0.244 - 1.000).  Fatigue: in arm A significant lower fatigue grade 3 than in B: A vs. B: 2 % vs. 20 % (p = 0.020)  No significant adverse event related to 6-gingerol was observed.  **8:** Adherence: 98.4 % (A: 99.1 %; B: 97.7 %) |
| Li (2018) | RCT | N = 146  Cancer type: lung cancer  Cth: cisplatin-based regimens  Antiemetic therapy: 5-HT3  receptor antagonist, ondansetron, dexamethasone, aprepitant  Drop out: 6 | A: ginger capsules 2 x 250 mg per day  B: placebo  Duration: 1 cycle | 1. Incidence and severity of acute CINV  2. Incidence and severity of delayed CINV  3. QoL  4. Adherence  5. Success of blinding  6. Adverse events | **1:** No significant differences between the groups in the incidence of acute nausea (p = 0.174) and vomiting (p = 0.309).  No significant differences between the groups in mean nausea scores (p = 0.246) and frequency of vomiting (p = 0.256) in acute phase; no differences even after controlling for intake of aprepitant  **2:** No significant differences between the groups in the incidence of delayed nausea (p = 0.214) and vomiting (p = 0.813).  No significant differences between the groups in mean nausea scores (p = 0.347) and frequency of vomiting (p = 0.718) in delayed phase  **3:** No significant differences between the groups (p = 0.884)  **4:** Arm B: 5 participants took fewer capsules than the protocol required (6 - 8 out of 10)  **5:** Participants were likely to correctly guess their treatment arm (p = 0.01); given reasons: taste of ginger (A: 42.3 %; B: 27.5 %; p = 0.068), effect of the capsule (A: 21.1 %, B:10.1 %; p = 0.103)  **6:** No significant differences between the groups:  drowsiness (p = 0.163), dry mouth (p = 0.086), heartburn (p = 0.494), flushing (p = 0.184); no study- treatment-related adverse event was observed |
| Lua (2015)  Salihah (2016) | RCT | N = 75  Cancer type: breast cancer  Cth: different Cth agents (on highly emetogenic Cth = 86.7 %; low emetogenic = 13.3 %)  Antiemetic therapy: granisetron + dexemethasone + metoclopramide  Drop out: 15 | A: aromatherapy necklace filled with 2 drops ginger essential oil  B: fragrance-matched artificial placebo  Duration: 2 cycles | Lua:  1. Nausea (severity)  Vomiting (incidence)  2. Impact of CINV on patients’ HRQoL  Salihah:  3. Dietary intake  4. General perception of aromatherapy administration  Lua + Salihah:  5. Adverse events  6. Success of blinding | **1:** Day 1: in arm A significant lower severity of nausea than in arm B: 38.98 ± 21.65 vs. 42.95 ± 24.82 (p = 0.040)  Incidence of vomiting: no significant differences between the groups  **2:** HRQoL total score: arm A significantly superior to arm B: MD 4.44, 95 % CI 1.13 to 7.76, p < 0.001  Subcategories: arm A is superior to arm B in terms of role functioning (MD 1.94, 95 % CI -5.35 to 9.24, p = 0.001), fatigue (MD -3.15, 95 % CI -8.06 to 1.77, p = 0.002), nausea and vomiting (MD -3.61, 95 % CI -7.51 to 0.29, p < 0.001), pain (MD -2.78, 95 % CI -6.59 to 1.03, p = 0.017), loss of appetite (MD -6.67, 95 % CI -12.50 to -0.83, p < 0.001), constipation (MD -1.67, 95 % CI -6.25 to 2.92, p = 0.046).  The domains role functioning and loss of appetite reached a minimal clinically significant change (10 points from baseline levels).  **3:** Mean energy intake: significant main effect time and group; p's < 0.001, no significant interaction group x time (p = 0.638); at day 3 and 5: arm A significantly higher than arm B: kcal (Mean ± SD) A vs. B: day 3: 1262.5 ± 239.6 vs. 1237.8 ± 251.9 (p = 0.015)  day 5: 1424.9 ± 233.4 vs. 1392.5 ± 235.0 (p = 0.002)  Energy needs met: significant main effect time and group; p's < 0.001, no significant interaction group x time (p = 0.604);  at day 3 and 5, arm A significantly higher than arm B: in % (Mean ± SD) A vs. B: day 3: 77.9 ± 14.3 vs. 76.3 ± 14.5 (p = 0.010)  day 5: 87.8 ± 13.7 vs. 85.9 ± 13.9 (p = 0.002)  Ratios of carbohydrate, protein, fat, fiber: no significant differences between the groups (p = 0.111 - 0.924).  **4:** No significant differences between the groups  **5:** Lua: very mild dizziness or light-headedness (A: 1)  Salihah: not reported  **6:** Lua: 93.3 % effectiveness  Salihah: not reported |
| Manusirivithaya (2004) | RCT | N = 48  Cancer type: gynecological cancer  Cth: cisplatin  Antiemetic therapy: metoclopramide, dexamethasone, lorazepam  Drop out: 5 | A: ginger capsules  day 1: 2 x 250 mg  day 2 - 5: 4 x 250 mg  B: placebo  Duration: 2 cycles | 1. Acute nausea and vomiting (≤ 24 h)  2. Delayed nausea and vomiting (day 2 - 5)  3. Adverse events  4. Preference for one or the other antiemetic regimen | **1:** No significant differences between the groups (nausea: p = 0.875; vomiting: p = 0.754)  **2:** No significant differences between the groups (nausea: p = 0.294 - 0.865; vomiting: p = 1.000)  **3:** No significant differences between the groups (p = 0.109 - 1.000)  Reported adverse events: restlessness, diarrhea, constipation, headache, dizziness, heartburn, palpitations, akathisia, acute dystonic reaction  **4:** No preference: 41.9 %, regimen A: 39.5 %, regimen B: 18.6 % (p < 0.001) |
| Marx (2017) | RCT | N = 51  Cancer type: any type of cancer  Cth: moderately or  highly-emetogenic Cth  Antiemetic therapy: “standard therapy”  Drop out: 17 | A: ginger capsules 4 x 300 mg per day  B: placebo  Duration: 3 cycles | 1. CINV-related QoL  2. CINV-symptoms: nausea (acute, delayed), vomiting, retching  3. Nutritional status  4. Global cancer-related QoL  5. Cancer-related fatigue  6. CINV-prognostic factors (prevalence): age, gender, anticipatory CINV, Cth emetogenicity  7. Adherence  8. Quality of patient blinding  9. Adverse events | **1:** Cycle 1: arm A significantly superior to B, clinical relevance minimal.  Nausea-related QoL (median (25 th, 75 th percentiles)) A vs. B: 124.5 (113.2, 126) vs. 111 (99, 126) (p = 0.029)  Total CINV-related QoL (median (25 th, 75 th percentiles)) A vs. B: 124.5 (113.2, 126) vs. 111 (99, 126) (p = 0.043)  Cycle 2, 3 and for vomiting-related QoL: no significant differences between the groups (p > 0.05)  **2:** No significant differences in prevalence and severity of CINV in all cycles (p > 0.05).  Subgroup analysis: there was no significant group difference between participants with and without aprepitant (p > 0.05).  **3:** No significant differences between the groups in all cycles (p > 0.05)  **4:** Arm A significantly superior, clinically relevant:  cycle 1 (Mean ± SD) A vs. B: 85.1 ± 18.9 vs. 71.9 ± 18.3 (p = 0.015)  cycle 3 (Mean ± SD) A vs. B: 83.6 ± 15.0 vs. 75.1 ± 13.9 (p = 0.040)  No significant differences between the groups in cycle 2 (p = 0.077)  **5:** Arm A significantly superior, clinically relevant:  cycle 1 (Mean ± SD) A vs. B: 41.8 ± 13 vs. 32.2 ± 10.8 (p = 0.006)  cycle 3 (MW ± SD) A vs. B: 42.4 ± 10.2 vs. 36.1 ± 7.2 (p = 0.013)  No significant differences between the groups in cycle 2 (p = 0.23)  **6:** No significant associations (p > 0.05)  **7:** Min. 3 capsules/day: 71 % A: 67 %, B: 74 %  **8:** Assignment correctly guessed A vs. B: 63 % vs. 30 %  given reasons: taste of ginger (13 %), smell of capsules (20 %), lack of nausea (60 %)  **9:** Significant adverse events A vs. B: 1 vs. 3 participants, none assigned to intervention |
| Montazeri (2013) | RCT | N = 44  Cancer type: any type of cancer  Cth: 50 - 100 mg cisplatin  Antiemetic therapy: granisetron + dexamethasone, if necessary additionally metoclopramide  Drop out: 13 | A: ginger capsules (250 mg) 2 x 2 per day  B: placebo  Duration: 2 cycles | 1. Nausea (frequency, severity)  2. Vomiting / retching (frequency, severity) | **1:** Severity of nausea: results are not presented in an understandable way. 24 h after Cth: in arm A significantly lower severity of nausea than in B: MD -0.93 ± 3.39 vs. 1.80 ± 2.33; p = 0.01  Frequency of nausea: no significant differences between the groups  **2:** Severity of vomiting / retching: results are not presented in an understandable way.  Frequency of vomiting / retching: no significant differences between the groups |
| Panahi (2012) | RCT | N = 100  Cancer type: women with cancer (especially advanced breast cancer)  Cth: docetaxel, epirubicin, cyclophosphamide  Antiemetic therapy: granisetron + dexamethasone  Drop out: 22 | A: ginger capsules 3 x 500 mg per day  B: no additional intervention  Duration: 1 cycle | 1. Prevalence of nausea, vomiting and retching  2. Severity of nausea, vomiting and retching  3. Proportion of participants with no / mild / moderate / severe symptoms of nausea, vomiting, and retching.  4. Adverse events | **1:** Prevalence of nausea: 6 - 24 h after Cth: in arm A significantly lower than in arm B: 35.1 % vs. 58.5 % (p = 0.04)  Prevalence of vomiting and retching: no significant differences between the groups (p = 0.26 - 0.80)  **2:** No significant differences between the groups (p = 0.31 - 0.98)  **3:** No significant differences between the groups (p = 0.26 - 1.00)  **4:** Arm A: heartburn, headache, and vertigo |
| Ryan (2012) | RCT | N = 744  Cancer type: any type of cancer  Cth: not reported  Antiemetic therapy: 5-HT3 receptor antagonists + dexamethasone  Drop out: 168 | Ginger capsules (250 mg)  A: 2 x 3 per day  B: 2 x 2 per day  C: 2 x 1 per day  D: placebo  Duration: 3 cycles (first cycle: baseline) | 1. Severity of acute nausea (mean, maximum)  2. Delayed nausea  3. Anticipatory nausea  4. QoL  5. Vomiting  Additionally collected by the questionnaire, but not defined as an endpoint in the article:  6. Adverse events | **1:** Mean acute nausea: A - C vs. D (Change, SE): -0.350, -0.140 (p = 0.013); significant single difference D vs. C (p = 0.046), no significant differences for other individual comparisons (D vs. B, p = 0.076; D vs. A, p = 0.738).  Maximum acute nausea: A - C vs. D (Change, SE): -0.470, 0.160 (p = 0.003); significant single difference D vs. B (p = 0.036), D vs. C (p = 0.017), no significant differences for D vs. A (p = 0.431).  **2:** No significant differences between the groups  **3:** Tests of fixed effects showed that anticipatory nausea (p < 0.0001) is a factor in acute CIN  **4:** No significant differences between the groups  **5:** No significant differences between the groups  **6:** 9 x related to intervention (out of 24 total): gastrointestinal symptoms, such as stage 2 heartburn, bruising/flushing and rash, which led to drop out of affected individuals |
| Sanaati (2016) | RCT | N = 65  Cancer type: Breast cancer  Cth: not reported  Antiemetic therapy: dexamethasone, metoclopramide, aprepitant  Drop out: 20 | A: ginger capsules 2 x 500 mg per day  B: chamomile  C: no additional intervention  Duration: 1 cycle | 1. CINV (frequency, intensity)  Additionally collected by the questionnaire, but not defined as an endpoint in the article:  2. Probable use of other antiemetics  3. Missed cases of capsule taking  4. Adverse events | **1:** Intensity of nausea: no significant differences between the groups (p = 0.238)  Frequency of nausea: significant effects for arm A (p = 0.013), with significant difference between arm A and B (p = 0.002) and arm A and C (p = 0.006)  Frequency of vomiting: arm A and B significant effects (p < 0.0001; p = 0.02), no significant group difference between arm A and B (p = 0.177), but significant differences between arm A and C (p > 0.0001) and B and C (p = 0.003)  **2-4:** Not reported |
| Santos (2023) | RCT | N = 48  Cancer type: uterine cervical neoplasm  Cth: cisplatin (40 mg/m²) associated with radiotherapy  Antiemetic therapy: ondansetron  with dexamethasone  Drop out: 4 | A: ginger 2 x 500 mg per day  B: ginger 2 x 250 mg per day  C: placebo  Duration: 6 weeks | 1. Nausea (number of episodes: acute (24h after Cth), delayed (day 4); severity)  2. Vomiting (number of episodes: acute (24h after Cth), delayed (day 4); severity)  3. Symptoms associated with dehydration (incidence of dry mouth, feebleness, thirst, dizziness, dark urine) | **1:** Number of episodes for acute nausea: no significant differences between arm A and C (p = 0.40) and arm B and C (p = 0.93).  Number of episodes for delayed nausea: no significant change over the 6 weeks (p = 0.87) between the 3 groups (p = 0.28, p = 0.21).  Severity: in arm A and B more participants experienced a lower grade than in arm C: A (grade 0, 1, 2): 48.2%, 39.3%, 12.5%; B (grade 0, 1, 2): 49.3%, 33.3%, 17.4%; C (grade 0, 1, 2): 23.5%, 58.8%, 17.6%, but no p-value was given.  **2:** Number of episodes for acute and delayed vomiting: no significant change over the 6 weeks (acute: p = 0.35; delayed: p = 0.54) between the 3 groups (acute: p = 0.85, p = 0.54; delayed: p = 0.56, p = 0.28).  Severity: in arm A and B more participants experienced grade 0 than in arm C: A (grade 0, 1, 2): 75%, 21.4%, 3.6%; B (grade 0, 1, 2): 59.4%, 36.2%, 4.3%; C (grade 0, 1, 2): 54.4%, 44.1%, 1.5%, but no p-value was given.  **3:** Results are not presented in an understandable way. |
| Shokri (2017) | RCT | N = 49  Cancer type: ovarian cancer  Cth: platinum-based adjuvant Cth (carboplatin 5 - 6 AUC, paclitaxel 175 mg/m2)  Drop out: 0 | A: ginger capsules 2 x 1 g per day  B: placebo  Duration: 6 cycles | 1. Poor 12-month outcome (serum CA125 level >35U, radiologic evidence of metastases and recurrence or death).  2. 12-month disease-free survival  3. Side effects of Cth | **1:** Serum CA125 level: no significant group difference at all time points (p = 0.80)  In relation to abnormally elevated CA125 level: no significant group differences (p = 0.24)  Metastases on CT scan:  T0 A vs. B: 45 % vs. 72.4 % (p = 0.05)  T2 A vs. B: 25 % vs. 55.2 % (p = 0.04)  No significant group difference at other time points (p = 0.18 - 0.49).  Mortality: no significant differences between the groups (p = 0.68)  Adverse prognoses: significantly less frequent in arm A than in B: OR = 3.3 p = 0.04  **2:** No significant differences between the groups (p = 0.55)  **3:** No significant differences between the groups.  Reported side effects: nausea and vomiting (p = 0.57), weight loss (p = 0.66), peripheral neuropathy (p = 0.58), bone marrow depression (p = 0.54), transient cortical blindness (p = 0.41), other side effects (p = 0.11). No intervention-related adverse events |
| Shooriabi (2016) | RCT | N = 40  Cancer type: head neck cancer  Cth: radiotherapy, no Cth  Drop out: 0 | A: ginger capsules 4 x 500 mg per day  B: placebo  Duration: 2 weeks | 1. Xerostomia symptoms (dry mouth, difficulty chewing/swallowing/speaking, oral burning sensation, bad taste, nightly awakening, grid denture, failure to meet people, non-speaking to people, no leaving the house)  2. Saliva quantity (weight, unstimulated)  3. Satisfaction with intervention (improvement in xerostomia, reduction in bad taste, removing of nightly xerostomia, removing nightly awakenings, medication use, improving stuck denture, quality of life, desire to meet people, leave home, higher association to people, ease of using medication, impact on xerostomia, feeling better) | **1:** Arm A: significant improvement in terms of dry mouth (general, daily, nightly), difficulty chewing/swallowing/talking, oral burning sensation, nightly awakening, bad taste, saliva weight (p < 0.05)  Arm B: significant improvement in terms of: dry mouth, difficulty chewing / swallowing, oral burning sensation (general, nightly), meeting people, saliva weight (p < 0.05)  **2:** Significantly higher increase in arm A than in B: (Mean ± SD) A vs. B: 2.051 ± 0.659 vs. 0.997 ± 0.947 (p < 0.01)  **3:** Arm A: improvement in medication use: yes vs. no: 0.75 vs. 0.25 (p = 0.041)  Positive impact on stuck denture: yes vs. no: 0.75 vs. 0.25 (p = 0.041)  Arm B: positive influence on stuck denture: yes vs. no: 0.85 vs. 0.15 (p = 0.003)  Increased desire to leave the house: yes vs. no:  0.75 vs. 0.25 (p = 0.041)  Ease of using medication/feeling better (unclear indicated which of the two factors the reported outcome refers to): yes vs. no: 0.25 vs. 0.75 (p = 0.041)  Authors indicate that in terms of tend to use medication and positive impact on stuck denture, arm A is significantly superior to B. |
| Sontakke (2003) | RCT | N = 60  Cancer type: any type of cancer except malignancies  of GIT  Cth: cyclophosphamide 500 - 1000 mg  Antiemetic therapy: no additional therapy  Drop out: 10 | A: ginger capsules (500 mg) 2 x 2 per day  B: metoclopramid  C: ondansetron  Duration: 3 cycles | 1. Nausea (none = complete control; mild to moderate = partial control; severe = no control)  2. Incidence of vomiting (no vomiting = complete control; 1 - 2 episodes = partial control; ≥ 3 episodes = no control)  3. Adverse events | **1:** Complete control: A vs. B vs. C: 62 % vs. 58 % vs. 86 %; C significantly higher (p < 0.01), no significant difference between A and B.  Partial / no control: no significant differences between the groups  **2:** Complete control: A vs. B vs. C: A vs. B vs. C: 68 % vs. 64 % vs. 86 %; C significantly higher (p < 0.01), no significant difference between A and B.  Partial / no control: no significant differences between the groups  Subgroup analysis regarding complete control of vomiting:  Patients with low dose Cth + cytotoxics causing mild emesis: no significant differences between the groups  Patients with low dose Cth + cytotoxics causing severe emesis: A vs. B vs. C: 14.3 % vs. 15 % vs. 29.5 %; C significantly higher (p < 0.01), no significant difference between A and B.  Patients with high dose Cth + cytotoxics: A vs. B vs. C: 58.3 % vs. 54.1 % vs. 87.5 %; C significantly higher (p < 0.01), no significant difference between A and B.  Incidence of vomiting 0 – 12 h after Cth: A vs. B vs. C: 10 vs. 9 vs. 3; 12 – 24 h after Cth: A vs. B vs. C: 20 vs. 19 vs. 6; in all three groups, the incidence of vomiting was significantly higher after 12 – 24 h than after 0 – 12 h (p < 0.05).  **3:** Reported adverse events: oral ulcers (3), alopecia (5), diarrhea (2). No group assignment and no group comparison reported. No adverse effects attributable to ginger were recorded. |
| Thamlikitkul (2017) | RCT | N = 34  Cancer type: breast cancer  Cth: adriamycin-cyclophosphamide regimen  Antiemetic therapy: ondansetron + dexamethasone, if necessary: ondansetron + domperidone or metoclopramide  Drop out: 0 | A: ginger capsules 2 x 500 mg per day  B: placebo  Duration: 2 cycles | 1. Reduction in nausea score  2. Vomiting (incidence)  3. Rate of rescue  medication use  4. Incidence of chemotherapy dose reduction or  Delay  5. Adverse events | **1:** No significant differences between the groups for acute (p = 0.64), delayed (p = 0.21) nausea and during the first five days of Cth (p = 0.30)  **2:** No significant differences between the groups (p = 0.5)  **3:** No significant differences between the groups (p = 0.4)  **4:** Dose reduction: arm A vs. B: 3 % vs. 3 %  Delay: arm A vs. B: 9 % vs. 6 %  **5:** No significant differences between the groups. Reported adverse events: fever, fatigue, mucositis, diarrhea, constipation, neutropenia, thrombocytopenia |
| Uthaipaisanwong (2020) | RCT | N = 48  Cancer type: gynecological cancer  Cth: carboplatin-paclitaxel  Antiemetic therapy: dexamethasone, ondansetron, ranitidine,  if needed, dimenhydrinate  Drop out: 1 | A: ginger capsules 4 x 500 mg per day  B: placebo  Duration: 2 cycles | 1. Nausea score  2. Vomiting (frequency)  3. Nausea grade  4. Adverse events | **1:** Day 1 (acute): in arm A significant lower mean nausea score than in arm B (Mean ± SD): 0.96 ± 1.57 vs. 1.49 ± 2.3 (p = 0.03)  Day 2 - 5 (delayed): no significant differences between the groups (p = 0.39 – 0.94)  **2:** No significant differences between the groups (acute: p = 0.78; delayed: p > 0.05)  **3:** No significant differences between the groups (p > 0.05)  **4:** No significant differences between the groups:  diarrhea (p = 0.34), constipation (p = 0.09), heartburn (p = 0.38) |
| Yekta (2012) | RCT | N = 98  Cancer type: breast Cancer  Cth: single day Cth  Antiemetic therapy: kytril or granisetron hydrochloride + dexamethasone  Drop out: 18 | A: ginger capsules 4 x 250 mg per day  B: placebo  Duration: 1 cycle | 1. Vomiting: anticipatory (72 h before Cth), acute (up to 24 h after Cth), delayed (24 - 72 h after Cth), total  2. Adverse events (anticipatory, acute, delayed) | **1:** Vomiting cases were significantly lower in arm A at anticipatory (A vs. B (Min, Mean ± SD, Max): 0, 0.05 ± 0.3, 2 vs. 0, 1.5 ± 5.9, 35; p = 0.04), acute (A vs. B (Min, Mean ± SD, Max): 0, 2.7 ± 1.2, 15 vs. 0, 3.7 ± 2.5, 17; p = 0.04), and delayed (A vs. B (Min, Mean ± SD, Max): 0, 3.3 ± 1.1, 18 vs. 0, 7.9 ± 3.9, 37; p = 0.003) phases and total sum of 6 days (A vs. B (Min, Mean ± SD, Max): 0, 2.3 ± 5.1, 23 vs. 0, 7.9 ± 14, 54; p = 0.002)  **2:** No significant differences between the groups at anticipatory (p = 0.2), acute (p = 0.06), and delayed (p = 0.5) phases.  Reported adverse event: heartburn |
| Zick (2009) | RCT | N = 162  Cancer type: any type of cancer  Cth: various Cths  Antiemetic therapy: 5-HT3 receptor antagonists with aprepitant if necessary  Drop out: 0 | Ginger capsules (250 mg)  A: 2 x 4 per day  B: 2 x 2 per day  C: placebo  Duration: 1 cycle | 1. Prevalence and severity of delayed CINV (> 24 h after Cth)  2. Prevalence and severity of acute CINV (≤ 24 h after Cth)  3. Adverse events / safety  4. Assessment of blinding | **1:** Prevalence of delayed nausea: no significant differences between the groups (p = 0.15 respectively p = 0.16).  Prevalence of delayed vomiting: no significant differences between the groups (p = 0.35 respectively p = 0.07).  Severity of delayed nausea: no significant differences between the groups regarding patients without the use of aprepitant (p = 0.69). Regarding patients who used aprepitant arm A and B experienced significantly higher severity of delayed nausea than arm C: A: 3.9 ± 0.9; B: 2.9 ± 1.3, C: 2.2 ± 0.7 (p = 0.01). Single tests show that arm A experienced significantly higher severity than B and C: ger (Mean ± SD): C = 2.8 ± 1.2, B = 2.9 ± 1.1, A = 3.4 ± 1.1 (p = 0.03).  Severity of delayed vomiting: no significant differences between the groups (p = 0.88 respectively p = 0.77).  **2:** Prevalence of acute nausea: no significant differences between the groups (p = 0.76 respectively p = 0.68).  Prevalence of acute vomiting: no significant differences between the groups (p = 0.35 respectively p = 0.47).  Severity of acute nausea: no significant differences between the groups (p = 0.47 respectively p = 0.55).  Severity of acute vomiting: no significant differences between the groups (p = 0.61 respectively p = 0.91).  **3:** No significant differences between the groups regarding adverse events in total, non-serious adverse events, serious adverse events (p = 0.07), dyspnea, gastrointestinal events, laboratory abnormalities (p = 0.06).  In arm C were significantly more fatigue (A: 0; B: 1; C: 5; p = 0.03) and miscellaneous adverse events (A: 1; B: 3; C: 8; p = 0.02) than in A and B.  **4:** Investigators could not guess allocation (p = 0.27), patients could guess allocation significantly correctly (p = 0.01).  Given reasons: taste of ginger (A: 33 %; B: 25 %, C: 9 %; p = 0.01), effect of the capsule (A: 33 %, B: 51 %, C: 16 %; p = 0.12) |
| Cth: chemotherapy; CINV: chemotherapy induced nausea and vomiting; CIN: chemotherapy induced nausea; QoL: quality of life; HRQoL: health-related quality of life; PG-SGA: Patient-Generated Subjective Global Assessment | | | | | |

### Table e3: Excluded studies

| **Reference** | **Year** | **Title** | **Journal** | **Reason for Exclusion** |
| --- | --- | --- | --- | --- |
| Sanatani, M.; Younus, J.; Stitt, L.; Malik, N. | 2015 | Tolerability of the combination of ginger (*Zingiber officinalis*), gentian (*Gentiana lutea*) and turmeric (*Curcuma longa*) in patients with cancer-associated anorexia | Journal of Complementary and Integrative Medicine | No RCT |
| Hickok, J. T.; Roscoe, J. A.; Morrow, G. R.; Ryan, J. L. | 2007 | A phase II/III randomized, placebo-controlled, double-blind clinical trial of ginger (*Zingiber officinale*) for nausea caused by chemotherapy for cancer: A currently accruing URCC CCOP Cancer Control study | Supportive Cancer Therapy | Study protocol of Ryan 2012 |
| Muthia, R; Wahyu, W | 2013 | Effect of ginger infusion on chemotherapy induced nausea and vomiting in breast cancer patients | J Biol Agr Healthcare | Not randomized |
| Alparslan, G. B.; Ozkarman, A.; Eskin, N.; Yilmaz, S.; Akay, M.; Acikgoz, A.; Orsal, O. | 2012 | Effect of Ginger on Chemotherapy-Induced Nausea and/or Vomiting in Cancer Patients | Journal of the australian traditional-medicine society | Not randomized |
| Crichton, M.; Marshall, S.; Isenring, E.; Lohning, A.; Koh, A.; McCarthy, A. L.; Molassiotis, A.; Bird, R.; McPherson, I.; Shannon, C.; Marx, W. | 2022 | Ginger supplementation a safe and viable adjuvant to antiemetic medications for chemotherapy-induced nausea and vomiting: a randomized trial | Supportive Care in Cancer | Only abstract available |

### Table e4: Risk of Bias table

| **Reference** | **Study type** | **Standardized rating of risk of bias** | **Additional comments on methodology** |
| --- | --- | --- | --- |
| Ansari (2016) | RCT | RoB  Domain 1: low  Domain 2: high  Domain 3: high  Domain 4: low  Domain 5: low  **Overall risk of bias: high** | CONTRA:  no ethics vote  Methodical quality: no power analysis; no ITT analysis; no control for multiple testing; no test of comparability of groups; blinding not tested  Report quality: no information about randomization; no information on how adverse events or adherence were collected; no indication of at what time points data were collected or when intervention started; barely any information on baseline characteristics; no significance values given in the comparisons; no accurate indication of drop-out reasons for other than "form not completed"; overall low report quality |
| Arslan (2015) | RCT | RoB  Domain 1: some concerns  Domain 2: high  Domain 3: high  Domain 4: some concerns  Domain 5: low  **Overall risk of bias: high** | PRO:  ethics vote  Methodical quality: power analysis  CONTRA:  Methodical quality: randomization by alternating allocation; no blinding  Report quality: no precise indication whether final evaluated number of participants also matches the number of randomized participants; adherence not assessed or reported; no information on adverse events collection; no time period specified; no information on whether reported data were collected in one or two cycles; no registry reported |
| Bossi (2017) | RCT | RoB  Domain 1: some concerns  Domain 2: high  Domain 3: high  Domain 4: low  Domain 5: low  **Overall risk of bias: high** | PRO:  ethics vote  Methodical quality: power analysis; no difference in drop-out rate between groups; groups comparable at baseline (according to authors, no p-values).  CONTRA:  Methodical quality: no ITT analysis; blinding not tested; reasons for dropout differ between groups - possibly lack of blinding; high dropout rate (39 %); different Cth not evaluated separately  Report quality: adherence not assessed or reported |
| Chenbing (2023) | RCT | RoB  Domain 1: low  Domain 2: some concerns  Domain 3: high  Domain 4: low  Domain 5: low  **Overall risk of bias: high** | PRO:  ethics vote  Methodical quality: power analysis; groups comparable at baseline  CONTRA:  Methodical quality: only single blinded  Report quality: adherence not assessed or reported; no precise information on antiemetic therapy |
| Crichton (2024) | RCT | RoB  Domain 1: low  Domain 2: some concerns  Domain 3: low  Domain 4: low  Domain 5: low  **Overall risk of bias: some concerns** | PRO:  ethics vote  Methodical quality: power analysis; ITT analysis; adherence assessed (pill count); testing of blinding  CONTRA:  Methodical quality: number of participants calculated by power analysis was not reached; blinding was not successful in 34% of the participants in the intervention group  Report quality: no detailed information on the Cth the patients received |
| Fahimi (2011) | RCT | RoB  Domain 1: some concerns  Domain 2: some concerns  Domain 3: some concerns  Domain 4: low  Domain 5: low  **Overall risk of bias: some concerns** | PRO:  ethics vote  Methodical quality: sample size in reference to other study  Report quality: information on the validation of the measurement instrument  CONTRA:  Methodical quality: no ITT analysis; blinding not tested; no survey of adverse events; no specification of cut-off level for too low compliance and thus exclusion from analysis; no comparison of baseline characteristics  Report quality: no information on randomization; no information on patient adherence; no group size reported; no time period reported; no information of cancer stage, number of prior treatments or Cth cycles; drop-out due to pruritus and GIT complaints not stated whether in intervention group or control; no information on CoI or funding; no registration reported |
| Kadhim (2021) | RCT | RoB  Domain 1: some concerns  Domain 2: low  Domain 3: low  Domain 4: high  Domain 5: low  **Overall risk of bias: high** | PRO:  ethics vote  Report quality: information on the validation of the measurement instrument  CONTRA:  Methodical quality: no power analysis; randomization by alternate days - risk for bias, as not completely randomized; no correction for multiple testing; no blinding - open design may lead to bias in assessment of severity of nausea; no systematic survey of adverse events; no comparison of baseline characteristics (B descriptively younger); different Cth not evaluated separately; Cth patients and mixed therapy patients not evaluated separately; about half of all patients had neither acute nor delayed vomiting over the study and up to a third never had nausea - very small sample in comparisons that had symptoms, especially on endpoint “Severity of acute and delayed nausea”  Report quality: no information on patient adherence; unclear when surveys for "posttest" occurred; no precise information on antiemetic therapy; no precise information on the time of ingestion of the ginger tea; no registration reported |
| Konmun (2017) | RCT | RoB  Domain 1: some concerns  Domain 2: some concerns  Domain 3: low  Domain 4: low  Domain 5: low  **Overall risk of bias: some concerns** | PRO:  ethics vote  Methodical quality: power analysis; adherence assessed  Report quality: detailed information on baseline characteristics and data collection  CONTRA:  Methodical quality: no ITT analysis; blinding not tested; baseline differences for stage of cancer  Report quality: no registration reported |
| Li (2018) | RCT | RoB  Domain 1: low  Domain 2: some concerns  Domain 3: low  Domain 4: low  Domain 5: low  **Overall risk of bias: some concerns** | PRO:  ethics vote  Methodical quality: power analysis; adherence verified with in-person interview and questionnaire, and pill counting; comparison of drop-out rates (p = 0.677, ns.); comparability of groups to baseline  Report quality: information on the validation of the measurement instrument  CONTRA:  Methodical quality: no ITT analysis, explanation that no information was available posttrial; blinding not successful; assessment of adverse events by verbal self-report - no systematic survey  Report quality: no information on cancer stage, number of previous treatments or Cth cycles; drop-out described without time point, no information on cut-off level for too low compliance and therefore exclusion from analysis |
| Lua (2015) | RCT | RoB  Domain 1: some concerns  Domain 2: low  Domain 3: high  Domain 4: high  Domain 5: low  **Overall risk of bias: high** | PRO:  ethics vote  Methodical quality: power analysis; modified ITT; Bonferroni correction (for multiple testing); control for sequence effects; blinding tested (93.3 % successful)  Report quality: accurate data on cancer stages, Cth cycles  CONTRA:  Methodical quality: only single blinded (investigators knew group assignment); no systematic collection of adverse events (participants should report them on their own)  Report quality: no registration specified |
| Manusirivithaya (2004) | RCT | RoB  Domain 1: some concerns  Domain 2: some concerns  Domain 3: some concerns  Domain 4: low  Domain 5: low  **Overall risk of bias: some concerns** | PRO:  ethics vote  Methodical quality: no sequence effects  CONTRA:  Methodical quality: no power analysis; no ITT analysis; blinding not tested; no comparison of baseline characteristics  Report quality: no information on patient adherence; no time period specified; no indication of cancer stage, number of prior treatments, or Cth cycles; no registration specified |
| Marx (2017) | RCT | RoB  Domain 1: some concerns  Domain 2: low  Domain 3: some concerns  Domain 4: low  Domain 5: low  **Overall risk of bias: some concerns** | PRO:  ethics vote  Methodical quality: power analysis; ITT analysis; blinding tested; adherence assessed  Report quality: information of the validation of the measuring instrument; detailed description of the questionnaires  CONTRA:  Methodical quality: according to power analysis n = 82, but only 51 were randomized; blinding poor, group assignment was often guessed - most common reasons were lack of nausea, smell/taste of capsules, reflux; in the third cycle, many participants (33 %) dropped out of the study  Report quality: no precise indication of "standard antiemetic therapy" |
| Montazeri (2013) | RCT | RoB  Domain 1: some concerns  Domain 2: high  Domain 3: high  Domain 4: some concerns  Domain 5: high  **Overall risk of bias: high** | PRO:  approval of the "behavior committee"  Methodical quality: examination of sequence effects  CONTRA:  Methodical quality: no power analysis; no ITT analysis; blinding not tested; no differentiation of gag reflex and vomiting  Report quality: author's blinding unclear; adherence not assessed or reported; adverse events not assessed or reported; little data on baseline characteristics; no information on cancer stage; outcomes not fully reported (no accurate data at all time points); no information on CoI; no registration reported; overall low reporting quality, as well as non-transparent presentation of results (especially when there is no significance) |
| Panahi (2012) | RCT | RoB  Domain 1: some concerns  Domain 2: some concerns  Domain 3: low  Domain 4: some concerns  Domain 5: low  **Overall risk of bias: some concerns** | PRO:  ethics vote  Methodical quality: power analysis; comparison of drop-out rates (p = 0.33, ns.)  Report quality: information on the validation of the measurement instrument  CONTRA:  Methodical quality: no ITT analysis; randomization by alternate days - risk for bias as not completely randomized; no blinding  Report quality: no information on patient adherence; no reporting of baseline characteristics (stated groups comparable to baseline); no differentiation between symptoms for severity; no registration specified |
| Ryan (2012) | RCT | RoB  Domain 1: some concerns  Domain 2: some concerns  Domain 3: low  Domain 4: low  Domain 5: some concerns  **Overall risk of bias: some concerns** | PRO:  ethics vote  Methodical quality: large sample size; adherence assessed (pill counting); no differences in number of drop-outs between groups  CONTRA:  Methodical quality: no power analysis; planned ITT, but then not all patients included in baseline cycle were evaluated resp. more evaluated than cycle 2 completed; blinding not tested; different Cth not evaluated separately  Report quality: no explanation of double blinding; only graphical representation of delayed nausea outcomes; no reporting of individual mean values across days; no reporting of values (means, etc.) on QoL and vomiting; overall results difficult to understand due to few details of statistical values and mostly graphical presentation |
| Salihah (2016) | RCT | RoB  Domain 1: some concerns  Domain 2: low  Domain 3: high  Domain 4: some concerns  Domain 5: high  **Overall risk of bias: high** | PRO:  ethics vote  Methodical quality: power analysis; modified ITT; Bonferroni correction (for multiple testing); control for sequence effects; blinding tested  Report quality: accurate data on cancer stages, Cth cycles  CONTRA:  Methodical quality: only single blinded (investigators knew group assignment); no systematic collection of adverse events (participants should report them on their own)  Report quality: no precise information on "standard antiemetic therapy"; endpoints of blinding and adverse events not reported; no registration specified |
| Sanaati (2016) | RCT | RoB  Domain 1: low  Domain 2: high  Domain 3: high  Domain 4: low  Domain 5: low  **Overall risk of bias: high** | PRO:  ethics vote  Methodical quality: adherence assessed  CONTRA:  Methodical quality: no power analysis; no ITT analysis; incorrect use of T-test (dependent sample instead of independent sample); blinding not tested  Report quality: adherence not reported; adverse events not reported; no information on cancer stage; use of other antiemetics not reported; no direction given concerning the significant differences; no data on CoI; overall low report quality; results section incomprehensible |
| Santos (2023) | RCT | RoB  Domain 1: low  Domain 2: low  Domain 3: low  Domain 4: low  Domain 5: some concerns  **Overall risk of bias: some concerns** | PRO:  ethics vote  Methodical quality: power analysis; ITT analysis  CONTRA:  Methodical quality: blinding not tested  Report quality: only graphical representation of symptoms associated with dehydration; no p-values given for severity of CINV; scarce information on baseline characteristics of the participants; no precise information on cancer stage; adverse events not assessed or reported; adherence not assessed or reported; for intervention groups different terms are used throughout the publication |
| Shokri (2017) | RCT | RoB  Domain 1: low  Domain 2: low  Domain 3: low  Domain 4: low  Domain 5: low  **Overall risk of bias: low** | PRO:  ethics vote  Methodical quality: group comparison of baseline characteristics  Report quality: detailed information on baseline characteristics  CONTRA:  Methodical quality: no power analysis; blinding not tested; adherence not assessed  Report quality: overall inadequate and not transparent; target group size in protocol n = 90; no drop-out seems unrealistic |
| Shooriabi (2016) | RCT | RoB  Domain 1: low  Domain 2: high  Domain 3: low  Domain 4: low  Domain 5: low  **Overall risk of bias: high** | PRO:  ethics vote  Methodical quality: adherence assessed  CONTRA:  Methodical quality: no power analysis; blinding not tested; no group comparison of baseline characteristics  Report quality: adherence not reported; adverse events not assessed or reported; no precise information on study period; little information on baseline characteristics; no precise information on cancer stage; symptoms included in the endpoint xerostomia incompletely reported; no information on CoI / funding; overall low reporting quality |
| Sontakke (2003) | RCT | RoB  Domain 1: some concerns  Domain 2: some concerns  Domain 3: high  Domain 4: low  Domain 5: low  **Overall risk of bias: high** | PRO:  ethics vote  Methodical quality: power analysis  CONTRA:  Methodical quality: no ITT analysis; blinding not tested; no testing of sequence effects; drop-out due to poor antiemetic control in intervention group; adverse events due to subjective assessment - no systematic survey; no comparison of baseline characteristics  Report quality: no information reported on randomization; adherence not reported; no time period reported; little information provided on patient characteristics; no information on cancer (stage, etc.) or which Cth cycle patients were in; no information on CoI or funding; no registration reported; very superficial report presentation |
| Thamlikitkul (2017) | RCT | RoB  Domain 1: low  Domain 2: low  Domain 3: low  Domain 4: low  Domain 5: low  **Overall risk of bias: low** | PRO:  ethics vote  Methodical quality: power analysis; ITT analysis; adherence assessed  CONTRA:  Methodical quality: blinding not tested  Report quality: no data on CoI or funding |
| Uthaipaisanwong (2020) | RCT | RoB  Domain 1: low  Domain 2: some concerns  Domain 3: low  Domain 4: low  Domain 5: low  **Overall risk of bias: some concerns** | PRO:  ethics vote  Methodical quality: power analysis; no sequence effects; adherence verified with face-to-face interview  Report quality: accurate information on patient characteristics  CONTRA:  Methodical quality: no ITT analysis; blinding not tested; comparison of 5 days of intervention only, no comparisons of remaining 16 days of cycle; at baseline only about half had CINV, unclear if comparable in both groups, by end of study: at least one-third at each time point with grade 0 nausea and generally little vomiting  Report quality: no information on comparability of groups (no separate listing for groups); not reported how often rescue drug was used; drop-out described without time point |
| Yekta (2012) | RCT | RoB  Domain 1: some concerns  Domain 2: high  Domain 3: low  Domain 4: low  Domain 5: low  **Overall risk of bias: high** | PRO:  ethics vote  Methodical quality: power analysis; adherence assessed (part of questionnaire)  CONTRA:  Methodical quality: no ITT analysis; blinding not tested  Report quality: hardly any information on questionnaires used; no information on cancer (stage, etc.) or which Cth cycle patients were in or which Cth was received |
| Zick (2009) | RCT | RoB  Domain 1: some concerns  Domain 2: low  Domain 3: low  Domain 4: low  Domain 5: low  **Overall risk of bias: some concerns** | PRO:  ethics vote  Methodical quality: power analysis; large sample size; control for multiple testing for primary endpoint; adherence assessed (pill count); testing of blinding (both assessors and patients)  Report quality: information on the validation of the measurement instrument  CONTRA:  Methodical quality: blinding not successful in participants - most common reasons were effect and taste; different Cth not evaluated separately  Report quality: no explanation of how correction for multiple testing was done, and no reference in table |
| Domain 1: risk of bias arising from the randomization process; Domain 2: risk of bias due to deviations from the intended interventions; Domain 3: risk of bias due to missing outcome data; Domain 4: risk of bias in measurement of the outcome; Domain 5: risk of bias in selection of the reported result;  Cth: chemotherapy; CoI: conflicts of interests; ITT: intention to treat | | | |
